# High-resolution structures of human NHE6 and NHE9 elucidate endosomal ion and lipid interactions

**DOI:** 10.1038/s41467-026-77254-0

**Published:** 2026-09-03

**Authors:** Jesper S. Hansen, Ashley C. W. Pike, Gamma Chi, Gernot Wolf, Jeppe Tranberg-Jensen, Hannah Lichtmannegger, David Speedman, Alvaro Ingles-Prieto, Fabian Goericke, Helena Batoulis, Hartmut Beck, Rajini Rao, Tooraj Mirshahi, David B. Sauer, Giulio Superti-Furga, Kilian V. M. Huber

**Affiliations:** 1 Centre for Medicines Discovery, Nuffield Department of Medicine, University of Oxford, Oxford, UK; 2 Target Discovery Institute, University of Oxford, Oxford, UK; 3 CeMM Research Center for Molecular Medicine of the Austrian Academy of Sciences, Vienna, Austria; 4 Bayer AG, Pharmaceuticals, Research & Development, Wuppertal, Germany; 5 The Department of Physiology, The Johns Hopkins University School of Medicine, Baltimore, MD USA; 6 Department of Genomic Health, Geisinger, Danville, PA USA; 7 Center for Physiology and Pharmacology, Medical University of Vienna, Vienna, Austria; 8 Carl and Gerty Cori Institute for Molecular and Computational Metabolism of the Austrian Academy of Sciences, Graz, Austria

**Keywords:** Cryoelectron microscopy, Membrane proteins, Permeation and transport

## Abstract

Endosomal NHE6 (SLC9A6) and NHE9 (SLC9A9) transporters are essential for maintaining pH homeostasis within endosomes and their dysfunction has been linked to neurological and neurodegenerative disorders. NHE6 and NHE9 are widely considered to function as electroneutral exchangers that couple the export of protons to the import of sodium or potassium ions across cellular membranes, thereby forming the basis of proton leak pathways for internal pH balancing and fine-tuning. Among the 13 identified SLC9 family members, only NHE6 and NHE9 are targeted to endosomes. Despite their biological importance and therapeutic potential, the structural basis for their activity and regulation remains elusive. Here, we present the cryo-EM structures of human NHE9 and two splice variants of NHE6 that differ by alternative inclusion of the β-hairpin motif-containing loop domain located between transmembrane helices 2 and 3, showcasing structural diversity within the organellar NHE subfamily. By mapping the sodium-binding site, our results provide mechanistic insights into ion transport, and for NHE6 we provide evidence for a conserved PIP2-mediated regulatory mechanism. These findings provide a framework for understanding endosomal NHE function with implications for disorders such as Alzheimer’s disease and glioblastoma.

## Abstract (web summary)

NHE6 and NHE9 regulate endosomal pH and are linked to neurological disease. Here, the authors report cryo-EM structures of human NHE9 and two NHE6 splice isoforms, revealing sodium and lipid- binding sites, including an NHE6 phosphoinositide pocket.

## Introduction

Endosomes are membrane-bound compartments within cells that play crucial roles in protein and lipid sorting and trafficking^1^. The pH within these compartments progressively decreases as they mature, with early endosomes being less acidic than late endosomes and lysosomes^2^. This proton gradient is essential for the proper function of endosomes, influencing protein-protein interactions, enzymatic activity, and the separation of molecules for recycling or degradation. Endosomal pH is maintained by the competing activities of the vacuolar ATPase that actively pumps protons into the lumen and endosomally targeted sodium-proton exchangers (NHEs) that couple the export of a proton to the import of, primarily, Na^+^ and K^+^ to limit acidification in an electroneutral manner^3^. Especially within the central nervous system, NHEs are emerging as pharmacological targets, and their dysfunction has been linked to metabolic, neurologic, and neurodegenerative diseases as well as tumor progression^4–7^.

In humans, NHEs are encoded by thirteen genes of the SLC9 gene family^8^. Nine of those belong to the SLC9A gene subfamily, including NHE1-5, which are primarily active at the plasma membrane, whereas NHE6-9 are annotated as organellar proteins^9^. While NHE7 and NHE8 are associated with the Golgi apparatus, NHE6 and NHE9 are primarily found in endosomal compartments^10^.

NHE6 is encoded by the *SLC9A6* gene localized on the X chromosome. Expression is high in several insulin-responsive tissues including the liver, kidney, and adipose tissue^11^, but the protein is most abundant in the brain, particularly in neurons, where it is thought to participate in the targeting of intracellular vesicles and recycling of synaptic vesicles^12^. Loss-of-function mutations in *SLC9A6* result in the rare X-linked neurologic disorder Christianson syndrome^13,14^. While primarily affecting males, neurological issues are also observed in heterozygous females, who can develop phenotypes resembling corticobasal degeneration syndrome (CBDS) and atypical Parkinson’s disease (PD), particularly in later life^15–18^. Some female carriers with *SLC9A6* mutations also display detectable tau pathology, which is a hallmark of neurodegenerative diseases like Alzheimer’s disease (AD) and some forms of PD^19^. In late-onset AD, NHE6 expression is one of the most downregulated genes, and low protein abundance correlates with disease severity^20^. While evidence for the effect of NHE6 activity on amyloid plaque formation and clearance is conflicting, the data nevertheless suggest that modulating endosomal pH by targeting NHE6 could potentially be an avenue for AD prevention and treatment^21,22^.

Notably, multiple alternative mRNA splice variants of NHE6 exist, but studies have primarily focused on the two main isoforms, NHE6.0, the first characterized NHE6, and the canonical NHE6.1. At the protein level, a difference between the two isoforms is an additional 32-amino-acid insertion in NHE6.1 that forms a distinct loop domain comprising a beta-hairpin motif between helices 2 and 3 of the transmembrane domain (TMD). Given the distinctive conformation this extended loop adopts on dimerization^23^, we refer to it henceforth as the “crown” domain. This insertion is only found in NHEs that localize to intracellular organelles. The crown domain contains an additional glycosylation site and a cysteine residue capable of forming a disulfide bond that is not present in NHE6.0, though the functional significance of this region remains to be determined^24^.

The related NHE9 sodium-proton exchanger is encoded by the gene *SLC9A9*. The protein shares 62% amino acid identity with NHE6.1. One functional isoform of NHE9 exists and, like NHE6.1, contains the crown domain. Although the gene is autosomal and the protein is ubiquitously expressed, loss-of-function mutations in NHE9 have been linked to central nervous system-related disorders. These include attention deficit hyperactivity disorder (ADHD), autism spectrum disorder, and epilepsy, potentially by affecting synaptic transmission and neurotransmitter clearance^25^. In contrast, abnormal elevation of NHE9 activity has been linked to cancer progression, particularly glioblastomas, and hence inhibiting NHE9 could provide an unexplored potential target in therapeutic interventions for certain cancer types^26^.

Both NHE6 and NHE9 appear transiently at the cell surface after exit from the ER before being internalized^24^. Although NHE9 is enriched in inner ear hair bundle plasma membranes^27^, it mainly resides in recycling and late endosomes^28^. NHE6 is predominantly targeted to recycling and early endosomes^28^, but can undergo dynamic trafficking to the plasma membrane under certain stimuli, such as hypoxic conditions in cancer cells^29^ and during insulin stimulation in adipocytes^11^. Subcellular translocation of both NHE6 and NHE9 is mediated through a C-terminal binding site that interacts with receptor for activated C kinase 1 (RACK1)^30^, a scaffolding protein that is ubiquitously expressed in mammals and involved in diverse cell signaling pathways^31^. Although their roles at the plasma membrane remain under debate^23^, several studies have suggested that NHE6 and NHE9 activity is regulated by phospholipids, particularly phosphoinositides, such as phosphatidylinositol 4,5-bisphosphate (PI(4,5)P2), which is enriched at the plasma membrane, and phosphatidylinositol 3,5-bisphosphate (PI(3,5)P2) and phosphatidylinositol 3-phosphate (PI(3)P) which are predominant in endosomes^23,32–35^. However, the molecular basis of lipid binding remains enigmatic, and given the importance of NHE6 and NHE9 as potential therapeutic targets, a deeper understanding of their structures and regulation is needed.

Here, we determine cryo-electron microscopy (cryo-EM) structures of detergent-solubilised NHE9, NHE6.1, and NHE6.0, including co-structures with the regulatory lipid PIP2. These structures reveal distinct features of NHE6 and NHE9 that provide important mechanistic insights into endosomal sodium-proton exchange, PIP2-mediated regulation, and their potential as therapeutic targets.

## Results

### Cryo-EM structure of human NHE9

**Fig. 1:**
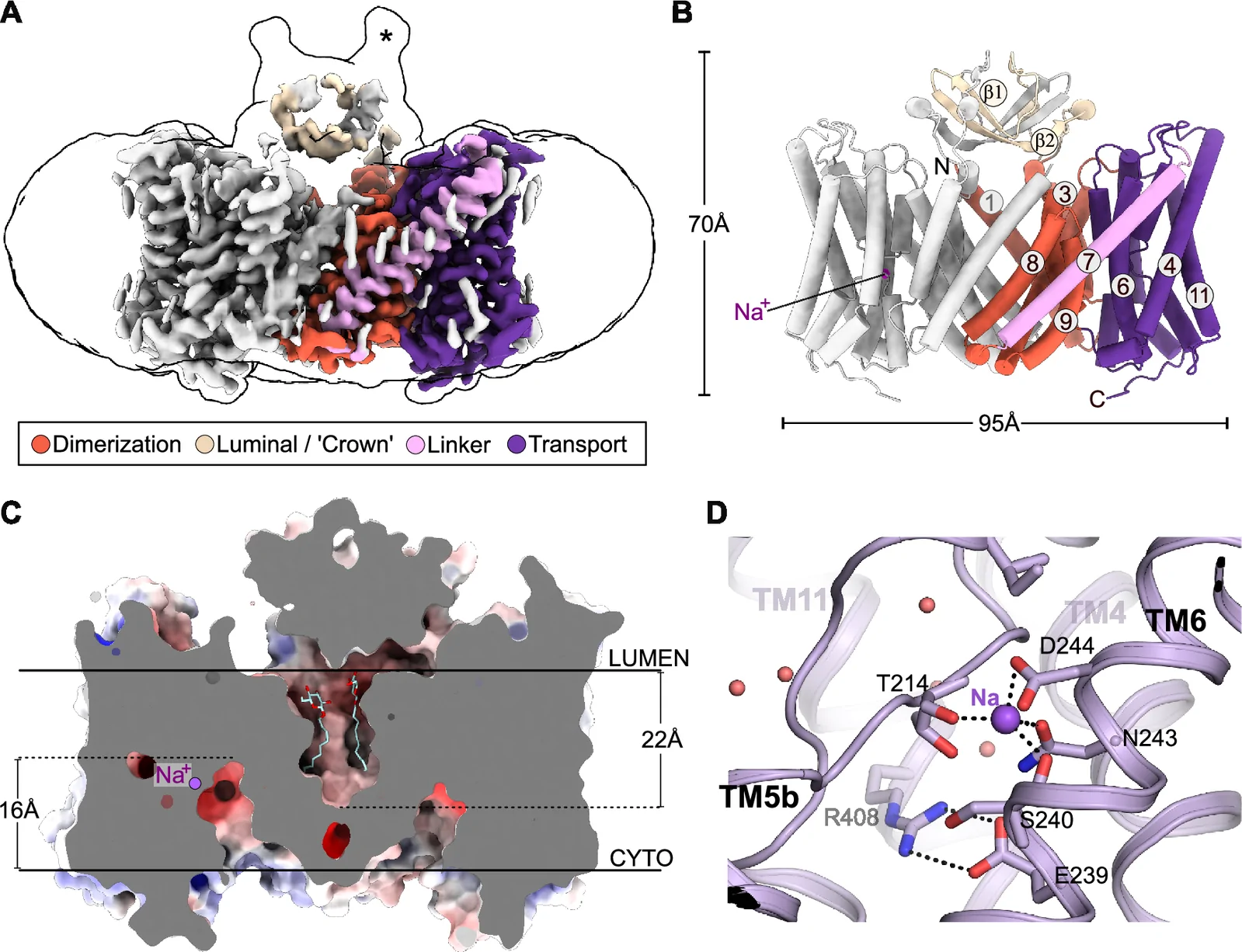
Sodium-bound structure of human NHE9. **A** Cryo-EM map. One NHE9 protomer is colored by functional region. A blurred map (black outline; applied B-factor of 100 Å^2^) highlights the extent of the detergent micelle as well as the crown domain. The putative glycosylation site (Asn96) in the crown domain is indicated by asterisk. **B** Protein structure of the NHE9 dimer. The structure is viewed as in (**A**) from the membrane plane. **C** Cutaway molecular surface view of NHE9 highlighting the inward-facing conformation. The molecular surface is colored by electrostatic potential contoured from −10 kT/e (red) to +10 kT/e (blue). Modeled detergent at the dimer interface is shown as sticks, and the position of a bound sodium ion is indicated. **D** Sodium binding site of NHE9.

We initially attempted to produce human NHEs using standard BacMam expression in Expi293 cells, but this strategy yielded broad peaks on size-exclusion chromatography unsuitable for structural and biochemical studies^36^. However, we observed high expression for a subset of NHEs while developing functional assays with SLC-expressing HEK293 Jump-In cells and during efforts to generate protein binders^37,38^. Immunofluorescence staining confirmed the expected subcellular punctate patterns consistent with NHE9 and NHE6 localization to endosomes (Supplementary Fig. 1). A subpopulation of each isoform was clearly detectable at the plasma membrane, consistent with their known transient localization to the cell surface. These data suggested that these cells could be used to inducibly express NHE6 and NHE9 via the integrated TetON promoter, enabling purification via the C-terminal TwinStrep tag. To facilitate large-scale protein production, we subsequently adapted the cells for suspension culture. We started with human NHE9, leveraging the previously characterized horse (*Equus caballus*) ortholog for validation of the purified protein^35^. Purifying NHE9 in DDM/CHS from the suspension-adapted cells after doxycycline induction yielded monodisperse and chemically homogenous protein. Cryo-EM analysis of this sample produced a high-resolution map at 2.6 Å (Fig. 1A, Supplementary Figs. 2, 3, and Supplementary Table 1), enabling unambiguous modeling of the protein along with tightly bound waters, ions, and lipids. Notably, no density was observed for an inhibitor added during purification.

In line with established models, the human NHE9 map revealed a dimeric structure of the transporter with a central scaffold domain and two peripheral transport domains. Although our construct used for protein purification was full-length (1–645), only residues Gln15-Asp494 were sufficiently resolved in the cryo-EM map for modeling. We hypothesise this is due to structural flexibility of the N- and C-termini, which has also been observed in the structures of NHE1 and NHE3^39,40^. The overall NHE9 structure resembles that of the horse orthologue^35^, forming a homodimer in which each protomer adopts the NhaA-fold characteristic for this family of sodium-proton exchangers with well-defined transport and scaffold domains^41^ The core TM1-TM13 domain of human and horse NHE9 is structurally similar (all atom RMSD 0.87 Å) with only subtle differences observed in the conformation of the luminal loops and a shift in the positioning of the short helix between TM3 and TM4 (Fig. 1B).

Thanks to the high resolution of the cryo-EM map, we were able to resolve a bound sodium ion within the active site of the transport domain. The transport domain is in the inward-facing conformation for both protomers, with the sodium binding site accessible from the cytoplasmic side of the transporter via a wedge-shaped tunnel situated between the transport and dimerization domains (Fig. 1C). The tunnel entrance is 24 Å × 10 Å wide and narrows to around 5 Å at the sodium site which lies 20 Å deep, away from the entrance (Fig. 1C). The sodium ion is coordinated by the main chain carbonyl groups of Thr214 on the TM5 interhelix region and Ser240 on TM6, as well as the side chains of Asn243 and Asp244 on TM6. The Na-O distances are between 2.16 and 2.5 Å, consistent with sodium coordination (Fig. 1D)^42^.

Analogous to the horse orthologue, the luminal crown domain (residues 72–120) of human NHE9 is poorly resolved with a local resolution of around 6 Å. However, the cryo-EM map prior to particle sieving provides a sufficiently resolved envelope to guide positioning and rigid-body refinement of the AlphaFold2-multimer model for this domain. Our map is consistent with the AlphaFold prediction that this region adopts a central beta-hairpin of β1 and β2, flanked by two short ɑ-helices on either side (Supplementary Fig. 3). In the dimer, the beta-hairpins from each protomer intertwine, with the β2 strands hydrogen-bonding with each other to form a partial 4-stranded beta barrel. Despite the limited resolution of the cryo-EM map in this region, the validity of the model is further supported by the positioning of Asn96 adjacent to a feature in the blurred map which likely corresponds to glycosylation of this residue. The crown domain floats above the core transmembrane domain, making minimal interactions with the rest of the transporter.

### Cryo-EM structures of two human NHE6 isoforms

**Fig. 2:**
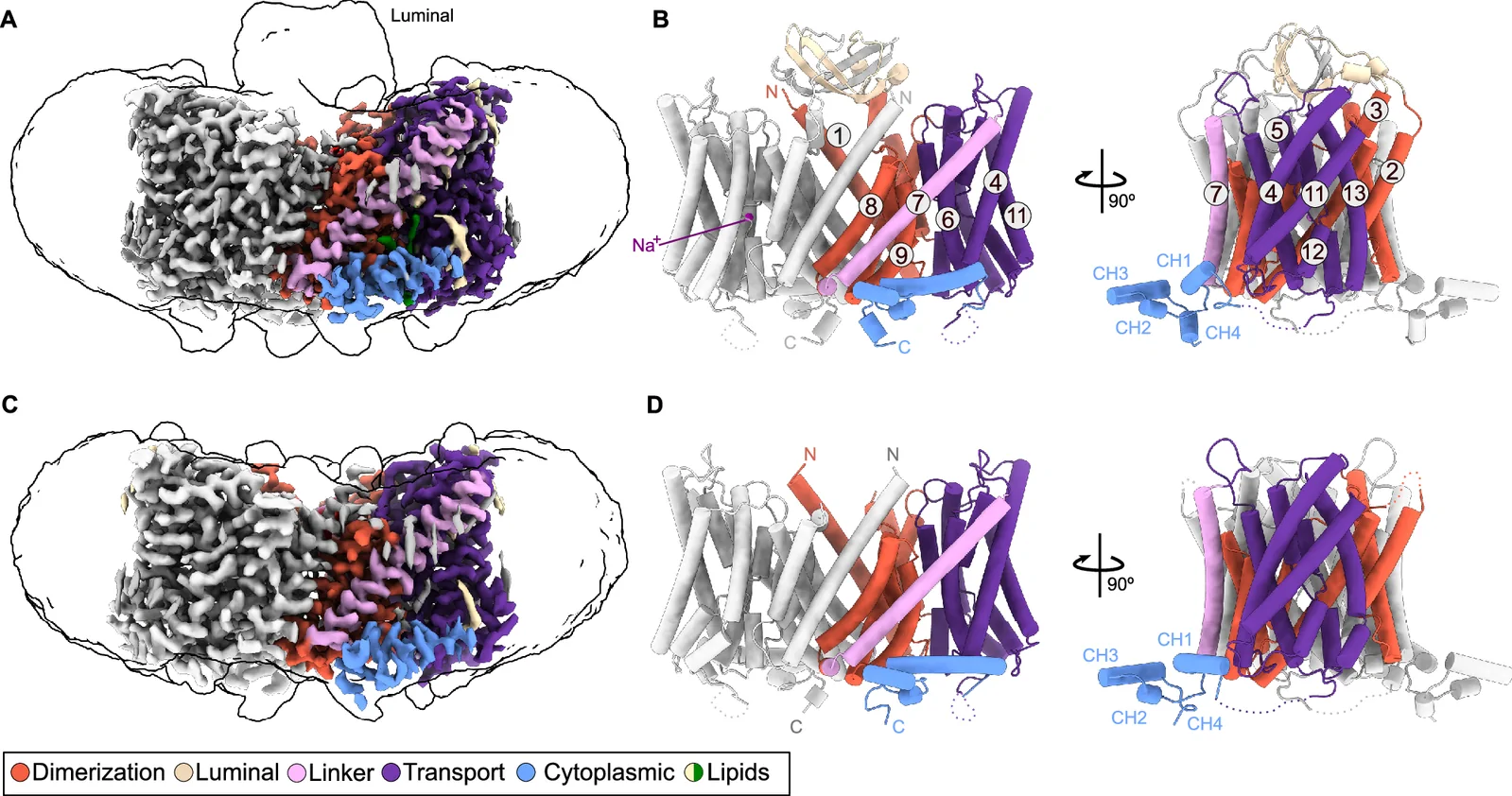
Structures of the two major isoforms of NHE6. Cryo-EM map for NHE6.1 (**A**) and NHE6.0 (**C**). The extent of the detergent micelle and luminal crown domain is highlighted by a *B*-factor blurred map (black outline; BF 100 Å^2^). Protein structures of NHE6.1 (**B**) and NHE6.0 (**D**). Perpendicular views are shown, and one protomer is colored by functional region.

Using an analogous expression, purification, and cryo-EM strategy, we next investigated the related human NHE6 proteins. This approach yielded structures of NHE6.1 at 2.33 Å resolution (Fig. 2A, B, and Supplementary Figs. 2, 4) and NHE6.0 at 3.2 Å (Fig. 2C, D, Supplementary Figs. 2, 5, and Supplementary Table 1). For NHE6.1, PI(3,5)P2 was included during sample preparation to resolve the binding sites for this regulatory lipid. Both NHE6 isoforms were captured in identical inward-facing conformations (NHE6/6.1/9 monomer core TM all atom RMSD 0.39–0.43 Å) (Supplementary Fig. 6), consistent with our DDM-solubilised NHE9 structure. This state appears to be the preferred conformation for purified NHE proteins in the absence of co-expressed auxiliary proteins or conformation-specific Fab fragments added during sample preparation^39,40,43^. For NHE6.1, the resolution of the cryo-EM map was sufficient to resolve the sodium site. Like NHE9, the sodium ion is coordinated by adjacent Thr, Ser, Asp, and Asn residues (Supplementary Fig. 7). The first residue visible in both NHE6 structures is Ser59 on the luminal/extracellular side, compared to residue position 15 in NHE9. This observation is consistent with previous data showing that NHE6 contains a cleavable signal peptide targeting NHE6 to the endoplasmic reticulum (ER) and subsequently to other cellular compartments like endosomes^44^. NHE9, lacking this signal peptide, is consequently targeted to distinct endosomal populations. Structurally, NHE6.1 and NHE6.0 are highly similar (dimer all atom RMSD of 0.548 Å). However, there are unique structural features exclusive to the respective isoforms. NHE6.1 has a 55-residue crown domain homologous to NHE9, encompassing residues 122–176, but this is less well resolved in NHE6.1, likely to indicate greater mobility. In contrast, the equivalent region in NHE6.0 is a significantly shorter 23-residue loop that is not resolved and is likely to be disordered. The dimer interface of the NHE6.1 isoform (4066 Å^2^) is slightly larger than NHE9 (3740 Å^2^). This is primarily due to the extended TM1 helix of NHE6.1 making additional interactions with the crown domain. The AlphaFold2-multimer model also predicts an interchain disulfide bond that connects the apex of the molecule in both NHE6.1 (Cys133) and NHE9 (Cys83) (Supplementary Fig. 3F). This prediction is supported by biochemical measurements which revealed a decrease (Δ*T*_m_ of ~1 °C) in thermostability upon addition of the reducing agent TCEP, capable of disulfide bond breaking, for both NHE6.1 and NHE9 (Supplementary Fig. 8). In contrast, NHE6.0 did not exhibit any change in thermostability under reducing conditions, consistent with the absence of a soluble domain with an interchain disulfide in this isoform.

A hydrophobic pocket formed at the interface between the homodimers has been suggested as critical for maintaining dimer stability (Fig. 1C)^23,35^. This structural feature is well-resolved in all our DDM-solubilised structures. From the luminal side, a 22 Å deep rectangular pocket is formed by TM1, TM3b, TM8, and TM10 at the dimer interface. This pocket is clearly occupied by non-protein densities that are consistent with two DDM molecules. These detergent molecules each interact with one NHE protomer, binding along the surface of TM3b and TM10 or between TM3b and TM8 (Supplementary Fig. 9).

### Cytoplasmic domain architecture of NHE6

**Fig. 3:**
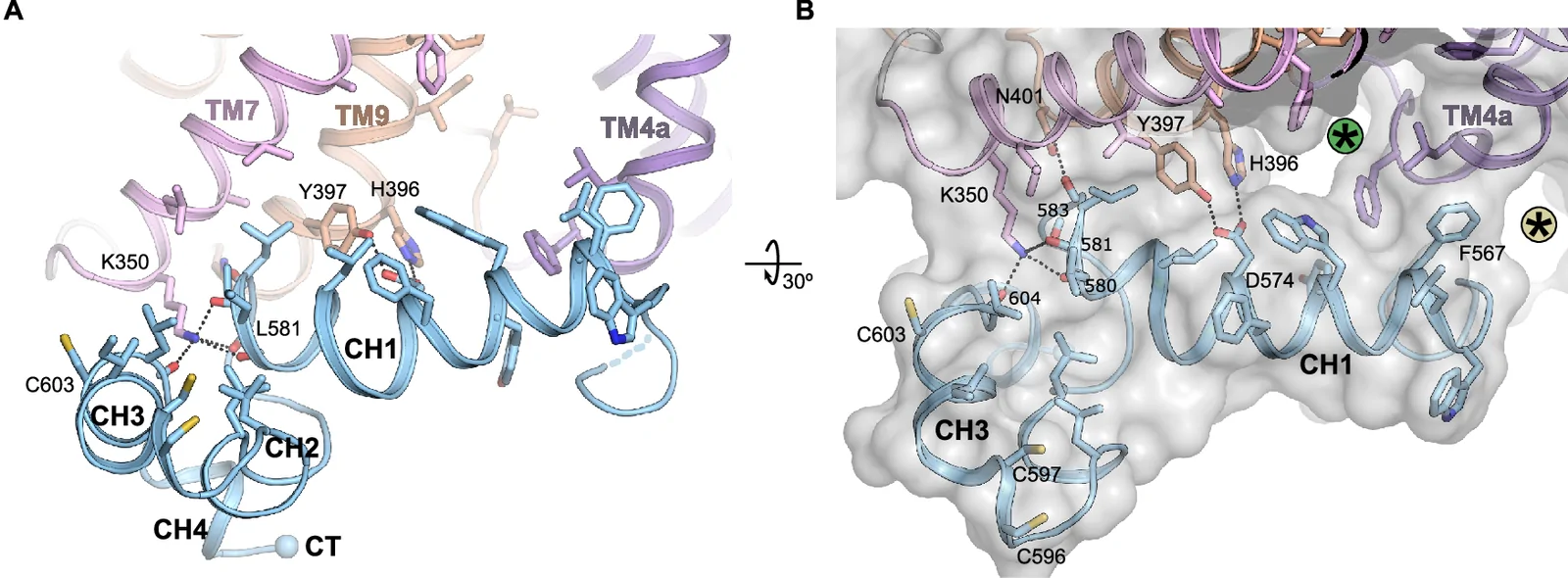
Cytoplasmic domain of NHE6. **A** The cytoplasmic domain (cyan). **B** Molecular surface representation highlighting interacting residues and two lipid-binding pockets. The positions occupied by the PIP2 and PL2 lipids are indicated by the colored asterisks (green: PIP2; wheat: PL2).

While the TMD is conserved among the different mammalian NHE isoforms, the C-terminal domain (CTD) exhibits significant sequence divergence that is thought to enable isoform-specific regulation (Supplementary Fig. 10). The scaffolding protein RACK1 is known to directly interact with the proximal CTD of organellar NHEs, including NHE6 and NHE9^30^. This proximal CTD is well resolved in both our NHE6 structures and adopts four C-terminal helices (CH1-4) (Fig. 2), collectively forming the binding site for RACK1. CH1 and CH3 lie parallel to the plane of the membrane, with sidechains from the amphipathic faces of each helix embedded into the detergent micelle (Fig. 3A). Multiple hydrophobic sidechains project up towards the inner leaflet from CH1, CH2, and CH3. In addition, three cysteine residues in the CH3 region may serve as palmitoylation sites to further stabilize the membrane association of this region^45^. The resolved proximal CTD region is rigidly attached to the TM core of the transporter via a network of hydrophilic interactions. The CTD ends of CH1 and CH3 helices are capped by the sidechain of Lys350 of TM7 which makes extensive interactions with the mainchain carbonyls of Pro580, Leu581, Thr583, and Leu604 (Fig. 3A). The alignment of CH1 is further stabilized by hydrogen bonds between Asp574 and His396 and Tyr397 on TM9 and an additional hydrogen bond between its C-terminus and the sidechain of Asn401 from TM7 (Fig. 3B). Overall, the interface between the TMD and the CTD buries about 650 Å^2^, though CH1 helix is kinked and bows away from the body of the transporter, creating a sizeable pocket between CH1, TM3, TM4, and TM9.

### Structures of NHE6 identify two phospholipid sites and a PIP2 binding pocket

**Fig. 4:**
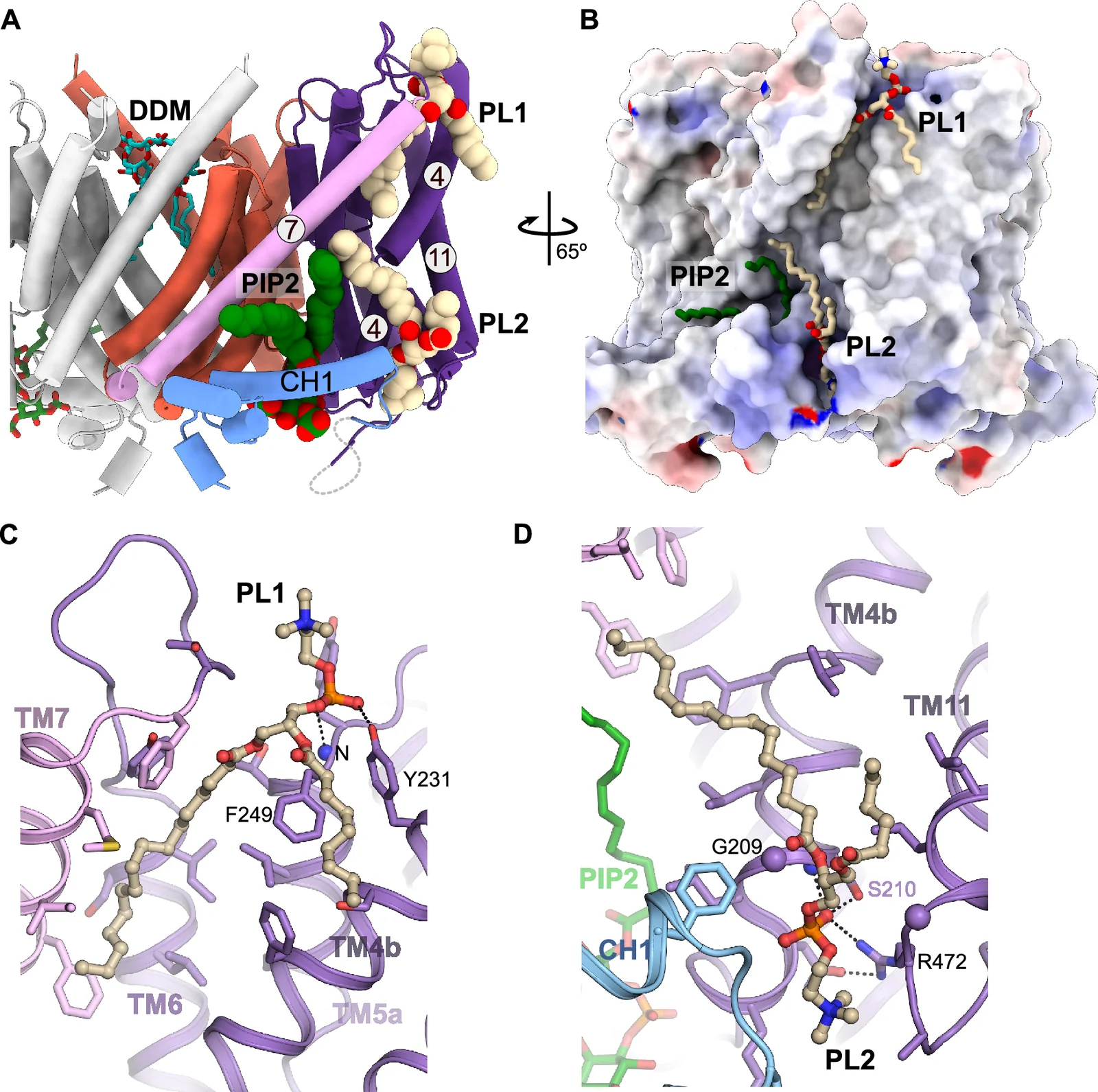
Phospholipid binding to NHE6. **A** Lipid sites on NHE6 monomer. The phospholipid (wheat) and PIP2 (green) are shown in sphere representation. **B** Molecular surface representation colored by electrostatic potential contoured from −10 kT/e (red) to +10 kT/e (blue). **C** PL1 site on luminal face. **D** PL2 site on cytoplasmic face.

Although all NHEs described here were obtained in detergent-solubilised form, we detected several well-resolved phospholipid densities evident in the cryo-EM maps (Fig. 4A). We identified two binding sites formed by narrow surface grooves that harbor well-resolved lipids, which are shared across all our NHE9 and NHE6 structures (Fig. 4B and Supplementary Fig. 11A–C, E). While the cryo-EM density was sufficient to model phospholipids at these sites, the identity of the lipids could not be precisely determined as the headgroups were less well resolved (see “Methods”). The first lipid, PL1, is located on the protein’s luminal face between TM7 and the adjacent transport domain (Fig. 4C). There is clear lipid-like density apparent for several acyl chains in this region. The “Y-shaped” PL1 density has one well-resolved acyl chain (18 Å in length) that runs parallel to TM7 in a crevice formed by TM6 and TM7. The lipid’s phosphate group interacts with the sidechain of Tyr231 and the backbone amide of Phe249, whose sidechain lies between the lipid’s two acyl chains. The second, less well-resolved acyl chain extends outward from the body of the transport domain toward the detergent micelle.

The second lipid site, PL2, is located on the cytoplasmic face of the transporter, buried between TM4 and TM11 of the transport domain and the CTD (Fig. 4D). Additional non-protein density is visible in this region in all NHE EM reconstructions reported here and, as with PL1, the density is “Y” shaped although the acyl chains are less well resolved. The modeled phosphate group engages the N-terminal end of TM4a and interacts with the sidechains of Ser210 from TM4b and Arg472 from TM11. One acyl chain lies diagonally across TM4b towards TM7 while the second is poorly resolved within the bulk micelle. This lipid binding pocket is also observed at the equivalent site in the human NHE3-CHP1 structure (Supplementary Fig. 11D, G–I) where it has been described as a bound phosphatidylinositol (PI)^40^. The consistent presence of PL1 and PL2 sites across NHE6, NHE9, and previously reported NHE structures strongly suggests that these represent structurally conserved lipid binding sites.

**Fig. 5:**
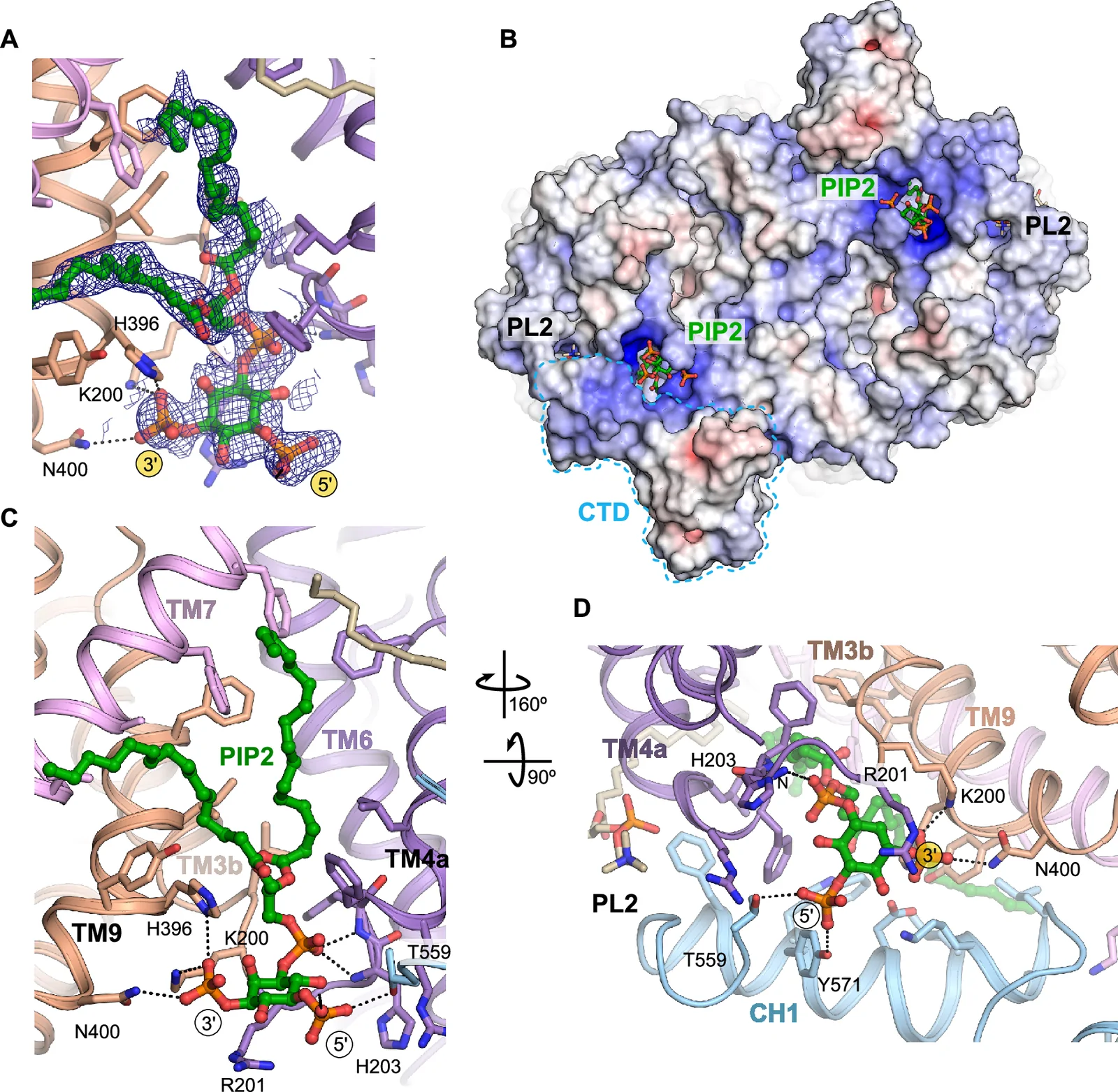
PIP2 binding to NHE6.1. **A** Cryo-EM density for bound PIP2, overlaid on the final NHE6.1 model. **B** Surface representation colored by electrostatic potential. The electrostatic surface potential map was calculated with APBS and is colored from −10 kT/e (red) through to 10 kT/e (blue). The view is oriented toward the cytoplasmic face of the transporter. Molecular details of PIP2 binding viewed parallel to the membrane plane (**C**) and from the cytoplasmic side (**D**).

To better resolve phosphoinositide-specific lipid binding sites, we supplemented purified NHE6.1 with the endosomally enriched PI(3,5)P2 during sample preparation. This regulatory lipid is clearly resolved in the cryo-EM map (Figs. 4A and 5A). The binding site for PI(3,5)P2 is located on the cytoplasmic face, within a narrow channel formed between the main body of the transporter and the cytoplasmic CTD CH1 helix. The cytoplasmic entrance to this channel is highly positively charged, complementing the negatively charged PI(3,5)P2 headgroup (Fig. 5B). A network of interactions coordinates the PIP headgroup, with the 3′ phosphate hydrogen-bonding with Lys200 from the loop between TM3 helices 3 and 4, and His396 and Asn400 from TM9, while the 5′ phosphate interacts with Thr559 and Tyr571 on CH1 (Fig. 5C). The backbone phosphate group is positioned at the N-terminal end of TM4a and makes interactions with the peptide amide groups of His203 and Phe204 (Fig. 5D). One of the fatty acid chains runs along a groove formed by TM4A and TM6 while the second runs across TM9 towards TM7. Several residues on the CH1 helix further contribute to coordinating the PIP2 headgroup. In the NHE6.0 structure prepared without PIP2 supplementation, this site adopts a similar conformation and appears to be partially occupied by an ordered, co-purifying lipid. However, the density is not sufficiently well resolved to allow definitive modeling. The similar structures of this region for NHE6.1 bound to PIP2, and NHE6.0 determined without added lipid, suggest that PIP2 binding does not cause any significant conformational change in the CTD. In contrast, in NHE9, residues 152–153 in the TM3/TM4 region adopt a helical conformation that is not compatible with PIP2 binding (Supplementary Fig. 11F).

The binding of NHE6s and NHE9 to PIPs was validated by in vitro binding experiments. Nano differential scanning fluorimetry (nanoDSF) measurements confirmed that PI(3,5)P2 stabilizes NHE6.1 (Δ*T*_m_ of ~4 °C), while PI(4,5)P2 exerted an even stronger effect (Δ*T*_m_ of ~7 °C) (Supplementary Fig. 8D). NHE6.0 and NHE9 displayed a similar preference for doubly-phosphorylated phosphoinositides, although the thermostabilizing effects were less pronounced for NHE9 compared to NHE6 (Supplementary Fig. 8D). This observation suggests that PIPs may bind to NHE9 at sites distinct from the identified NHE6-specific PIP2 site. Addition of PI(3)P to purified NHE6.1 did not result in significant thermostabilization, yet we observed modest effects for NHE6.0 and NHE9 (Supplementary Fig. 8D). These findings indicate isoform-specific differences for PI(3)P binding. Taken together, the data suggest that variations in PIP and PIP2 levels may contribute to the partially overlapping yet non-redundant functional roles of NHE6 and NHE9 in different cellular compartments.

## Discussion

Our results provide further structural insights into human NHE9 and NHE6 transporters, demonstrating both share a highly conserved ion coordination site. Although all our structures were captured in the inward-facing state, they are consistent with the gating and transport mechanisms proposed for the mammalian NHE family^35^. Our cryo-EM structure of NHE9 (2.6 Å) and NHE6.1 (2.33 Å) achieves sufficient resolution to unambiguously resolve a bound sodium within the ion binding site of the transport domain in contrast to earlier studies that inferred ion binding sites from structural and chemical homology to the archaeal PaNhaP^46^. Our high-resolution structures reveal the inward tunnel is open and solvent accessible with water molecules filling the cavity and region around the ion-binding site. Consistent with previous MD simulations of horse NHE9^35^, the bound sodium ion is coordinated by conserved Asp244, Asn243, Ser240, and Thr214 residues in human NHE9, or homologous residues in NHE6. In the MD simulations, sodium was only able to bind NHE9 when the equivalent of Asp244 was unprotonated. This agrees with the expected protonation state of aspartate side chains at our experimental pH of 7.5, though Asp244’s exact pKa may be modulated by the local environment^47^ and cannot be directly determined from our cryo-EM maps.

Based on our structural data and homologous sodium-proton exchangers, oligomerization of NHE6 and NHE9 to form homodimers is a prerequisite for their transport activity. Dimerization is facilitated through the scaffold domains of each protomer with interfaces involving TM helices. The luminal crown domain present in NHE6.1 and NHE9 makes additional contributions to their dimerization interface (Supplementary Fig. 6D). Notably, in our structural and biochemical data, NHE6.0 exhibits markedly reduced thermostability relative to NHE6.1 despite high sequence similarity between the two isoforms (Supplementary Fig. 8B). This suggests that the NHE6.1-specific crown domain’s additional 1848 Å^2^ of buried surface area and specific contacts with the transport domain underlie this increase in protein stability (Supplementary Fig. 6D). Western blot analysis of cell lysates revealed NHE6.1 is partially resistant to SDS, with monomer and dimer species, while NHE6.0 is primarily monomeric indicating it has a less stable dimeric configuration (Supplementary Fig. 8C). Furthermore, we found that paralog-specific differences determine thermostability, as NHE9 is similar to NHE6.1 in architecture and surface area at the dimer interface, yet is more thermostable and the dimeric species is SDS resistant. As membrane protein thermostability and oligomer resistance to SDS can correlate with in vivo protein localization or activity^48,49^, these in vitro biochemical differences might indicate distinct functional roles. We also observed phospholipid-dependent differences in thermostability. Similar to bacterial NapA, whose dimer retention is stabilized by cardiolipin^50^, annular lipids like PIP2 have been proposed to stabilize organellar NHE dimers^23,35^. In our NHE6 and NHE9 structures, the hydrophobic pocket created at the dimer interface is occupied by DDM molecules and consistent with a hydrophobic cavity at the luminal dimer interface capable of accommodating phospholipids. The uniformity of four DDM molecules filling the luminal hydrophobic pocket in all our structures suggests that the observed thermostabilization of NHE6.1, NHE6.0 and NHE9 by lipids is due to binding at distinct sites.

Doubly or triply phosphorylated phosphoinositides are known to differentially regulate NHE1’s transport activity, mediated by positively charged regions in the protein’s CTD that form electrostatic bonds with negatively charged lipids^32–34^. PI(4,5)P2 binding increases NHE1 transport activity and is regulated by competition with the inhibitory PI(3,4,5)P3 and possibly PI(3,5)P2^32^. Consistent with these observations, our structure of NHE6 revealed a phosphoinositide binding pocket that is sufficiently large to accommodate these multiple phosphorylation states of the PIP. Parallel work by Feilen et al. reported a 3.4 Å structure of human NHE6.1^51^. Similar to our NHE6.0 structure without added PIP2, this NHE6.1 map contains non-protein density consistent with ordered lipid within the CTD pocket. Our high-resolution structure of NHE6.1 with supplemented PI(3,5)P2 provides direct evidence that this site can bind PIP2 and reveals the previously unseen phospholipid binding sites PL1 and PL2. Our thermostability data corroborate that both PI(3,5)P2 and PI(4,5)P2 are able to bind to NHE6.1. The differential stabilizing effects of the various phosphoinositides on NHE6.0, NHE6.1, and NHE9 suggest differentiated regulatory roles of these lipids on these endosomal NHEs. The central role of NHE6 in regulating pH in early and recycling endosomes as well as at the plasma membrane by stimuli-induced recruitment from intercellular stores further supports a dual regulatory role of lipids. This may explain why NHE6 interacts with both endosomally enriched PI(3,5)P2 and plasma membrane-dominant PI(4,5)P2. By analogy to NHE1^33^, NHE6 activity can be modulated by these regulatory lipids. Hence, in addition to previously proposed roles of lipids to stabilize and regulate sodium-proton antiporters at the dimer interface, our results reveal PIP2 and other lipids binding NHE6 and NHE9 at the cytoplasmic face of the transport domain. However, this regulation may be complex as the lipid composition will vary by organelle and signaling processes, and the effects of each lipid may differ across NHE genes and the differing species of origin used in experimental studies.

Similar to NHE1, it is likely that NHE6 requires tight positioning of the proximal CTD region involved in RACK1-binding with the inner surface of the plasma membrane for optimal transport configuration. Notably, a structural feature that differs between NHE6 and NHE9 is the TM13-CH1 connecting loop, which is eight residues longer in NHE9. We hypothesize that this additional length increases flexibility, effectively decoupling the proximal CTD from the transmembrane core. This mobility likely reduces local order and thereby diminishes resolvability in the cryo-EM map under our sample conditions. Moreover, three cysteine residues, including a predicted palmitoylation site, are localized in the CH3 region of NHE6’s proximal CTD, and their sidechains project towards the membrane. Palmitoylation is the post-translationally reversible attachment of a C16 fatty acid to primarily Cys residues, which is crucial for regulating a vast array of cellular signalling processes, including facilitating PIP2 binding to proteins, including SLCs^52^. We did not observe density compatible with confident modeling of a palmitoyl group in our NHE6 structures, though this is not surprising due to the detergent-solubilized condition and the limitations of cryo-EM for resolving flexible post-translational lipidations. Nevertheless, it is tempting to speculate that the challenges in resolving the RACK1-binding region in NHE9 may be linked to stability differences in CTD regions, whereas NHE6 adopts a more stable configuration, possibly aided by palmitoylation, and hence allowing structure resolution.

The aberrant activity of NHE9 linked to glioblastoma progression and NHE6’s central role in neurodegenerative diseases like Alzheimer’s disease, and more recently, in adipocyte biology^53^, highlight these transporters as promising therapeutic targets. However, so far it has proven challenging to develop subtype-specific inhibitors for these organellar NHEs^54^. Therefore, the identification of a PIP2-specific site in NHE6 is interesting as it may represent a potential modulatory site that could be selectively targeted^55^. Such targeting might be achieved either through small molecule binders or manipulating downstream effectors to alter cellular PIP2 levels, thereby offering new avenues for therapeutic intervention.

## Methods

### Cell lines

Stable cell-lines with doxycycline-inducible expression of human full-length NHE9 (645 aa), NHE6.1 (701 aa), and NHE6.0 (669 aa) were generated by the RESOLUTE Consortium as previously described^56^. Briefly, codon-optimized cDNAs were cloned into a modified pJTI R4 CMV-TO MCS pA vector (Thermo Fisher Scientific) that contained a C-terminal HA-Twin-Strep tag. Jump-In T-REx HEK293 cells (Thermo Fisher Scientific) were stably transfected with these constructs as recommended by the manufacturer’s protocol. Transgene expression was induced via doxycycline treatment at 1 µg/ml for 24 h or the duration indicated. Parental Jump-In T-REx HEK293 cells were authenticated by STR profiling (Microsynth). Before analysis, the adherent HEK293JI-SLCx-SH cell lines were selected in DMEM medium supplemented with 10% v/v FBS, 100 µg/ml Pen/Strep, 5 μg/ml Blasticidin, and 2 mg/ml Geneticin for 2–3 passages and then cultured in DMEM supplemented with 10% v/v FBS and 100 µg/ml Pen/Strep at 37 °C and 5% CO_2_. Expression of the codon-optimised transgenes was verified by RNA sequencing, Western blotting, and immunofluorescence imaging. Plasmid constructs were verified by Sanger sequencing prior to cell line generation. No additional STR profiling or karyotyping was performed on the derived stable cell lines.

### Immunofluorescence

HEK293JI-SLCx-SH cells seeded (0.15 × 10^6^ cell density) in cell culture imaging dishes with a 35 mm diameter and a coverslip bottom (Ibidi GmbH) were induced by doxycycline at 1 µg/ml for 24 h. For plasma membrane staining, live cells were labeled using 7.5 µg/ml wheat germ agglutinin (WGA) Alexa Fluor 647 conjugate diluted in Hank’s balanced salt solution according to the manufacturer’s instructions (Invitrogen). All cells were fixed with 4% formaldehyde in phosphate-buffered saline (PBS) for 15 min, then permeabilized and blocked in blocking buffer consisting of PBS supplemented with 2% w/v fish gelatin and 0.1% w/v saponin for 1 h. For plasma membrane or nuclei co-staining, the permeabilized cells were incubated with mouse monoclonal Alexa Fluor 488 anti-HA.11 Epitope Tag [16B12] (Biolegend #901509, 1:1000) for 2 h at room temperature or overnight at 4 °C. For nuclei co-staining, cells were counterstained with SYTOX Deep Red Nucleic Acid Stain (Invitrogen) according to the manufacturer’s instructions. For co-immunolabelling, the permeabilized cells were incubated for 2 h at room temperature with the indicated primary antibody diluted in blocking buffer: HA (Biolegend #902301), 1:500; EEA1 (Proteintech #68065-1), 1:1000 and LAMP-1 (DSHB Cat# h4a3, RRID:AB_2296838), 1:200. The cells were then washed with PBS three times over 30 min and then incubated for 1 h with either Alexa Fluor 647-labeled goat anti-mouse IgG (Invitrogen #A-21236) or Alexa Fluor 488-labeled goat anti-rabbit IgG (Invitrogen #A-11034) antibodies diluted 1:800 in blocking buffer. Finally, cells were again washed with PBS three times over 30 min and then mounted with ProLong Glass Antifade Mountant (Invitrogen/Thermo Fisher Scientific). Imaging was performed on an LSM 710 laser-scanning confocal microscope system connected to a Zeiss AXIO Observer Z1 inverted microscope using an EC Plan-Neofluar 40/1.30 objective. Figures were prepared using FIJI (ImageJ v1.54p) to merge the pictures taken with different channels^57^.

### Western blotting

Cells cultured in T-25 flasks were harvested at sub confluency following 24 h doxycycline (1 µg/ml) treatment, washed in PBS, and cell pellets were flash-frozen in liquid nitrogen until use. Cell pellets were then thawed and lysed in RIPA lysis and extraction buffer (Thermo Scientific) supplemented with Protease Inhibitor Cocktail III (EMD Millipore, 1:1000) and Benzonase Nuclease (EMD Millipore, 1:1000 dilution of ≥250 units/μl solution). Lysates were centrifuged at 14,000 × *g* for 15 min (4 °C), and the protein concentration in the supernatant was determined by the Pierce BCA Protein Assay (Thermo Scientific). Lysates (40 µg) were then subjected to standard SDS-PAGE and blotted onto nitrocellulose membranes. The membrane was blocked for 1 h in PBS and 0.1% (w/v) Tween 20 (PBS-T) containing 6% (w/v) skimmed milk. The membrane was washed with PBS-T containing 2% (w/v) skimmed milk, and then probed with primary antibodies comprising polyclonal rabbit anti-HA.11 epitope Tag (BioLegend #902301, 1:1000) and monoclonal mouse β-Actin antibody (Sigma-Aldrich #A5441, 1:500) in PBS-T containing 2% (w/v) skimmed milk. Detection was performed with fluorescently-conjugated Alexa Fluor 680 goat anti-rabbit IgG (H+L) (Invitrogen #A-21076, 2 mg/ml, 1:5000) and Alexa Fluor 790 goat anti-mouse IgG (H+L) (Invitrogen #A11375, 2 mg/ml, 1:5000) using a LI-COR ODYSSEY CLx imager.

### Protein expression and purification

To produce recombinant NHE proteins, HEK293JI-SLCx-SH cells were first expanded adherently in T-175 flasks, then converted into suspension cell cultures by standard serum dilution in 2 l air-vented shaker flasks. Subsequently, cells were cultured at 37 °C, 175 rpm, 5% CO_2_ in FreeStyle293 medium. Protein expression of human full-length NHE9, NHE6.1, or NHE6.0 was induced at a cell density of 2 × 10^6^ cells/ml by addition of 1.5 µg/ml doxycycline to suspension-adapted HEK 293JI-SLCx-SH cell lines and cultured at 30 °C, 175 rpm, 5% CO_2_. After 72–96 h, the cells were harvested by centrifugation at 900 × *g* for 20 min, washed with PBS, and pelleted again prior to flash-freezing in liquid nitrogen, and then stored at −80 °C until use.

To purify overexpressed protein, the cell pellets were resuspended in Buffer A (50 mM HEPES pH 7.5, 200 mM NaCl, 5% glycerol) and lysed by mechanical disruption with two passes through an Emulsiflex C5 homogenizer (Avestin). The NHE proteins were extracted from cell membranes by addition of a 10:1 mixture of n-dodecyl-β-D-maltopyranoside (DDM)/cholesteryl hemisuccinate (CHS) to a final concentration of 1% (w/v) for DDM and 0.1% for CHS. The cell suspension was incubated at 4 °C for 1 h on a rotator (10 rpm) before centrifugation at 32,000 × *g* for 1 h at 4 °C to remove insoluble cell debris. The supernatant was aspirated, added a slurry of Buffer A pre-equilibrated 50% v/v Strep-Tactin XT Sepharose chromatography resin (Cytiva) at 2 ml per l original culture volume, and incubated at 4 °C on a rotator (6 rpm) for 2 h. The resin was collected by centrifugation at 700 × *g* for 5 min, washed three times with 10 column volumes of Buffer A supplemented with 0.03% w/v DDM/0.003% CHS, and transferred to a gravity column. The resin-bound proteins were eluted with 0.1 M D-Biotin (Chem-Impex) dissolved in Buffer A after adjusting to pH 7.5 and supplementing with 0.03% DDM/0.003% w/v CHS. Protein-containing fractions were pooled and concentrated to <1 ml using a 100 kDa MWCO Vivaspin concentrator centrifugal concentrator (GE Life Sciences) and further purified by size exclusion chromatography (SEC) on a Superose 6 Increase 10/300 GL size exclusion column (GE Life Sciences) in SEC buffer (20 mM HEPES pH 7.5, 200 mM NaCl, 0.015% DDM/ 0.0015% CHS).

### Protein thermostability measurements

For all thermostability measurements, proteins were diluted to a final concentration of 6 µM in SEC buffer. For reducing conditions, the proteins were incubated with 5 mM TCEP on ice for 1 h. To characterize phosphoinositide binding, stock solutions of the respective lipids, phosphatidylinositol 3-phosphate diC16 (PI(3)P; Echelon Biosciences cat no. P-3016), phosphatidylinositol 3,5-bisphosphate diC16 (PI(3,5)P2; Echelon Biosciences cat no. P-3516) and phosphatidylinositol 4,5-bisphosphate diC16 (PI(4,5)P2; Echelon Biosciences cat no. P-4516) were prepared with final concentration of 1 mg/ml in SEC buffer. The respective lipids were added to the purified proteins to a final concentration of 0.1 mg/ml and incubated for 1 h on ice. Subsequently, the protein samples were filled (10 µL) into Prometheus Standard Capillaries (NanoTemper). Melting curves were determined using Prometheus NT.48 NanoDifferential Scanning Fluorimetry (nanoDSF) by monitoring the intrinsic fluorescence at 350 nm relative to 330 nm during a temperature ramp with a rate of 1 °C/min from 20 to 95 °C. In vitro binding was conducted twice (*n* = 2 independent measurements). The melting temperature was determined from four technical replicate measurements for all samples, and the results were plotted using Prism 11.0.0 (GraphPad).

### Cryo-EM sample preparation

Grids were prepared by plasma-treating Quantifoil R1.2/1.3 mesh 200 or 300 gold grids and applying 3 µl freshly prepared protein sample at 4–5 mg/ml, followed by blotting for 7–9 s with blot force −5 and 20–30 s wait times at 100% humidity at 4 °C. Grids were then vitrified in liquid ethane, using a Vitrobot Mark IV (Thermo Fisher Scientific). For the NHE9 data, purified protein was incubated with the NHE inhibitor BAY-5784 (SMILES: ClC1=C(C=CC=C1)N2CCC(C3=C2C=C(N4C[C@@](N)(C)CCC4)C(F)=C3)=O) at 0.2 mM for 3 h at 4 °C, prior to protein concentration and grid preparation. The grids used for collecting this NHE6.1 data were prepared after adding 0.25 mM BAY-5784 and 10:1 mol/mol ratio of PI(3,5)P2 diC16 to protein and incubated for 3 h at 4 °C prior to grid preparation. Grids for NHE6.0 were prepared without the addition of additional lipids or inhibitors.

### Cryo-EM data acquisition and image processing

EM grids were screened on a 200 kV Glacios microscope prior to high-resolution imaging on a Titan Krios G3i microscope operating at 300 kV. In all cases, data were recorded using a Falcon 4i direct electron detector equipped with a SelectrisX energy filter using EPU software (Thermo Fisher) with a defocus range of −0.8 to −2.4 µm, energy filter slit width of 10 eV and a total exposure dose of 50 e^−^/Å^2^. (See Supplementary Table 1 for magnification, pixel size, and other collection details for the various datasets).

Data processing workflows are shown in Supplementary Figs. 3–5. The majority of the data processing was carried out in cryoSPARC (v3.3.1)^58^. Particles were exported to RELION (v4) using the PYEM Python package (v 0.5)^59^.

#### NHE9

The collected 11504 EER movies were grouped so that each frame experienced a dose of 1 e^−^/Å^2^ and motion-corrected in RELION with 5 × 5 patches. Corrected movies were imported into cryoSPARC for CTF correction (Supplementary Fig. 3). A subset of images was used for blob picking and 2D classification to generate templates to train a TOPAZ (v 0.2.4) picking model^60^. Subsequently, a total of 1,690,899 particles were identified from the full image set, extracted in a 384px box, and binned to a pixel size of 2.92 Å. The particle set was cleaned up using several rounds of 2D classification, resulting in a set of 773,749 particles which underwent 3D heterogenous refinement. The best set (557,760 particles) yielded an initial reconstruction with a GSFSC resolution of 3.29 Å. These particles were exported to RELION, re-extracted and binned to a pixel size of 1.09545 Å and subjected to static 3D classification (*K* = 10, *T* = 4) using a protein only mask, followed by Bayesian polishing and CTF refinement. These particles were subjected to further 3D static classification after re-refinement in cryoSPARC (*K* = 10, *T* = 8, protein mask). Particles from the best populated class (212,266 particles) were re-imported into cryoSPARC for final NU refinement, which generated a reconstruction with a GSFSC resolution of 2.83 Å with no symmetry imposed, and 2.72 Å with C2 symmetry applied. This C1 map was used for modeling and refining the full-length protein including the crown domain. The C1-refined particles also underwent further selection using CRYOSIEVE (v. 1.2.3)^61^. Each cycle of particle selection (“sieving”) was evaluated by reimporting particles from each CRYOSIEVE iteration back into cryoSPARC and monitoring final GSFSC resolution achieved after non-uniform (NU) refinement with C1 symmetry. Using this procedure, the “best” minimal subset of high-quality NHE9 particles (69556 particles) yielded a final cryo-EM map with a GSFSC resolution of 2.60 Å. This reconstruction was used to model the core TM region and more clearly highlighted the position of the sodium ion and some ordered solvent molecules.

#### NHE6.1

The 9002 movies collected were patch motion and CTF corrected in cryoSPARC (Supplementary Fig. 4). A subset of images was blob picked to obtain good quality templates for training a TOPAZ picking model. A total of 1,090,375 particles were extracted in a 320-pixel box and downsampled to a pixel size of 3.728 Å. After 2D classification, the remaining 586,700 particles were re-extracted in a box size of 360px at 0.932 Å/px and used to generate an ab initio model, which, after NU refinement gave an initial reconstruction of 2.64 Å with C2 symmetry imposed. Particles were subsequently corrected for local motion, and CTF global and per particle corrections were applied. These polished particles yielded a 2.55 Å map with no symmetry applied. Particles were further triaged with CRYOSIEVE using the same procedure as processing NHE9 to yield a reduced set of 153,800 particles that gave a final reconstruction after non-uniform refinement with a GSFSC resolution of 2.33 Å.

#### NHE6.0

The 6700 movies were motion-corrected using the eBIC on-the-fly auto processing pipeline in RELION with 5 × 5 patches (Supplementary Fig. 5). Patch CTF correction was applied in cryoSPARC. Particles picked using crYOLO (v1.9.6)^62^ were 2D classified to select a representative set of particles and used to train TOPAZ within cryoSPARC. A total of 1,001,670 particles were initially selected and extracted in a 384px box, downsampled to 128px at a pixel size of 2.763 Å. After 2D classification, 100,000 particles were used to generate 3 representative ab initio volumes that were used to classify all particles. The best resolved subset of particles was refined to give an initial 3.8 Å map with C2 symmetry imposed. Particles were re-extracted in a 256px box at 1.3815 Å/px and subjected to Bayesian polishing, and global and per-particle CTF refinement in RELION. Following re-refinement in cryoSPARC, the aligned, polished particles underwent 3D classification in RELION without alignment (10 classes, *T* = 4) using a mask covering the protein. Particles in the best class were re-imported into cryoSPARC for final non-uniform refinement, yielding a GSFSC resolution of 3.44 Å with C2 symmetry imposed and 3.59 Å without symmetry. The C1-refined particles underwent further selection using CRYOSIEVE, resulting in a subset of 52,384 particles that gave a final GSFSC resolution of 3.2 Å.

### Model building, refinement, and validation

In all cases, the initial model was fitted into the cryo-EM maps and manually adjusted with Coot (v. 0.9.8.93)^63^. The resulting model was then subjected to refinement with ISOLDE (v1.3)^64^ followed by phenix.real_space_refine (Phenix v. 1.21.1-5286) using secondary structure and loose reference model restraints to the input model. Ligand restraints were generated using Grade2 (v1.6.0)^65^. No restraints were used for the sodium site.

#### NHE9

An initial model derived from the AlphaFold database^66^ was fitted into the final cryoSPARC reconstruction obtained after particle filtering using CRYOSIEVE. In this map, the core TMD domain was well resolved, but the crown domain (73–120) was poorly defined (Supplementary Fig. 3A). Furthermore, the expected CTD region (495–645) was disordered. The model was adjusted manually in Coot and refined against the auto-sharpened map. The final NHE9 core model encompasses residues 15–494 excluding the crown domain, lipids PL1 and PL2, a sodium ion, 3 peripheral DDM molecules, and a handful of solvent molecules per monomer. Density at the dimer interface was modeled as four detergent DDM molecules based on the length of the acyl chains. In each case, the first glucose unit in the maltoside headgroup was reasonably well-resolved and has been included. While the PL1 and PL2 lipids were modeled as phosphatidylcholine (PC), their headgroup is not sufficiently resolved in the EM maps to categorically identify the precise lipid type. For the full-length model including the crown domain, the predicted NHE9 dimer was generated by AlphaFold2-multimer, and the crown domain of residues 69–123 was aligned within the cryo-EM map obtained prior to particle sieving based using the TMD helices TM2 and TM3 from the core TM model as anchor points. The cytoplasmic domain structure was then relaxed into the map, refined using ISOLDE, grafted onto the core TMD model, and further refined in Phenix using a map sharpened with a B-factor of −25 Å^2^. As the cryo-EM map for the crown was less well resolved, most sidechains for this region were truncated in the model. The interchain disulfide bridge between adjacent Cys83, predicted by AlphaFold, was preserved and appears compatible with the experimental map.

#### NHE6.0

The trimmed AlphaFold NHE6.0 model was initially fitted into the final 3.2 Å cryo-EM map obtained after particle sieving. As with NHE9, the sieved map has well resolved density for the core TM helices but, in addition, the proximal cytoplasmic domain of residues 528–578 was also apparent. The remaining 91 residues (579–669) at the C-terminus were disordered and not modeled. Manual adjustments were carried out in Coot, followed by refinement with ISOLDE and Phenix. The refined model comprises the entire protein chain for residues 59–578, apart from the poorly resolved loops between TM2-TM3 (122–140) and TM13-CH1 (512–527). In addition to the protein, well resolved lipids PL1 and PL2, and 2 DDM molecules were modeled per monomer. The PL1/PL2 lipids were modeled as phosphatidylethanolamine based on the size and shape of the headgroup. At 3.2 Å, discrete density for the sodium ion is unresolved (Supplementary Fig. 7C).

#### NHE6.1

The refined dimer model for the NHE6.0 short isoform was fitted into the NHE6.1 map obtained after particle sieving. In addition to the PL1 and PL2 lipids, well-defined cryo-EM density was observed for the added PIP2 at the cytoplasmic face of the transporter. Manual adjustments of the local structure around the PIP2 binding site were carried out using Coot. Well-ordered solvent molecules were added, as well as a Na^+^ ion in the Na site (Supplementary Fig. 7A). The final model for the core transporter comprises protein residues 59–121, 170–543, and 559–612 as well as one sodium ion, the three ordered lipids PL1, PL2, and PIP2, two DDM molecules, and 21 solvent molecules per monomer. As with NHE6.0, the distal C-terminal residues (613–701) were disordered. Overall, the cryo-EM density map for the cytoplasmic crown domain in NHE6.1 was less defined than NHE9, and local resolution estimation indicated that this region has a resolution of around 6 Å. As with NHE9, a dimeric model of the full-length NHE6.1 transporter was initially predicted using AlphaFold3. The crown domain from this structure was initially aligned using the TM2 and TM3 helices and then relaxed into the NHE6.1 cryo-EM 2.55 Å map obtained from all polished particles (blurred with an applied B-factor of 40 Å^2^) using ISOLDE. The crown domain region was then grafted onto the core TM model with most sidechains pruned to Cbeta and then refined in Phenix using Ramachandran and loose reference model restraints to the input coordinates.

Figures were prepared with ChimeraX v1.3^67^ and PyMOL (2.2.0)^68^.

### Reporting summary

Further information on research design is available in the Nature Portfolio Reporting Summary linked to this article.

## Supplementary information

Supplementary Information

Reporting Summary

Transparent Peer Review File

## Source data

Source Data

## Data Availability

The data that support this study are available from the corresponding authors upon request. The cryo-EM maps and models generated in this study have been deposited in the Electron Microscopy Data Bank (EMDB) and the Protein Data Bank (PDB), respectively. NHE9 core (EMD-53828PDB 9R8K), NHE9 including luminal crown domain (EMD-53827PDB 9R8J), NHE6.0 (EMD-53829PDB 9R8L), NHE6.1 core/PIP2 complex (EMD-53832PDB 9R8N), NHE6.1 including luminal crown domain/PIP2 complex, (EMD-53831PDB 9R8M). The horse NHE9 and human NHE1 and NHE3 used in this study for structural comparisons are available under accession codes 8PVR7DSV, and 7X2U, respectively. The protein detection, protein isolation, and biophysical characterization data generated in this study are provided in the Supplementary Information/Source data file. The Source data underlying Supplementary Figs. 2B, D, F, and 8 are provided as a Source data file. Source data are provided with this paper.
