# Supplementary material for: High-resolution structures of human NHE6 and NHE9 elucidate endosomal ion and lipid interactions
Source: Nat Commun. 2026 Sep 3;17:10478. doi: 10.1038/s41467-026-77254-0 (PMC13635234; doi:10.1038/s41467-026-77254-0)
Supplement: Supplementary file 1 [file 41467_2026_77254_MOESM1_ESM.pdf]

SUPPLEMENTARY INFORMATION

High-resolution structures of human NHE6 and NHE9 elucidate endosomal ion and lipid interactions

Jesper S. Hansen[1,2,‡], Ashley C. W. Pike[1,‡], Gamma Chi[1], Gernot Wolf[3], Jeppe Tranberg-Jensen[1,2], Hannah Lichtmannegger[1,2], David Speedman[1,2], Alvaro Ingles-Prieto[3], Fabian Goericke[4], Helena Batoulis[4], Hartmut Beck[4], Rajini Rao[5], Tooraj Mirshahi[6], David B. Sauer[1], Giulio Superti-Furga[3,7,8], Kilian V. M. Huber[1,2,*].

1. Centre for Medicines Discovery, Nuffield Department of Medicine, University of Oxford, Oxford, OX3 7FZ, UK.

2. Target Discovery Institute, University of Oxford, Oxford, OX3 7FZ, UK.

3. CeMM Research Center for Molecular Medicine of the Austrian Academy of Sciences, Vienna, Austria.

4. Bayer AG, Pharmaceuticals, Research & Development, 42113 Wuppertal, Germany.

5. The Department of Physiology, The Johns Hopkins University School of Medicine, Baltimore, MD, USA.

6. Department of Genomic Health, Geisinger, Danville, PA, USA.

7. Center for Physiology and Pharmacology, Medical University of Vienna, Vienna, Austria.

8. Carl and Gerty Cori Institute for Molecular and Computational Metabolism of the Austrian Academy of Sciences, Graz, Austria.

‡These authors contributed equally to this work.

*Corresponding author: Kilian V. M. Huber (kilian.huber@cmd.ox.ac.uk)

Contents:

Supplementary Figure 1 – Cellular distribution of NHE9 and NHE6 in HEK cells

Supplementary Figure 2 – NHE9 topology / NHE9, NHE6.1 and NHE6.0 protein production data

Supplementary Figure 3 – NHE9 cryo-EM data processing workflow

Supplementary Figure 4 – NHE6.1 cryo-EM data processing workflow

Supplementary Figure 5 – NHE6.0 cryo-EM data processing workflow

Supplementary Figure 6 – NHE structures

Supplementary Figure 7 – Cryo-EM density for sodium binding site

Supplementary Figure 8 – Thermostability of NHE6 and NHE9

Supplementary Figure 9 – Detergent molecules fill cavity at dimer interface

Supplementary Figure 10 – Sequence alignment for NHE9, NHE6.1, NHE6.0 and related sequences

Supplementary Figure 11 – Ordered lipid binding sites in NHEs

Supplementary Table 1 – Cryo-EM data collection, processing and validation statistics

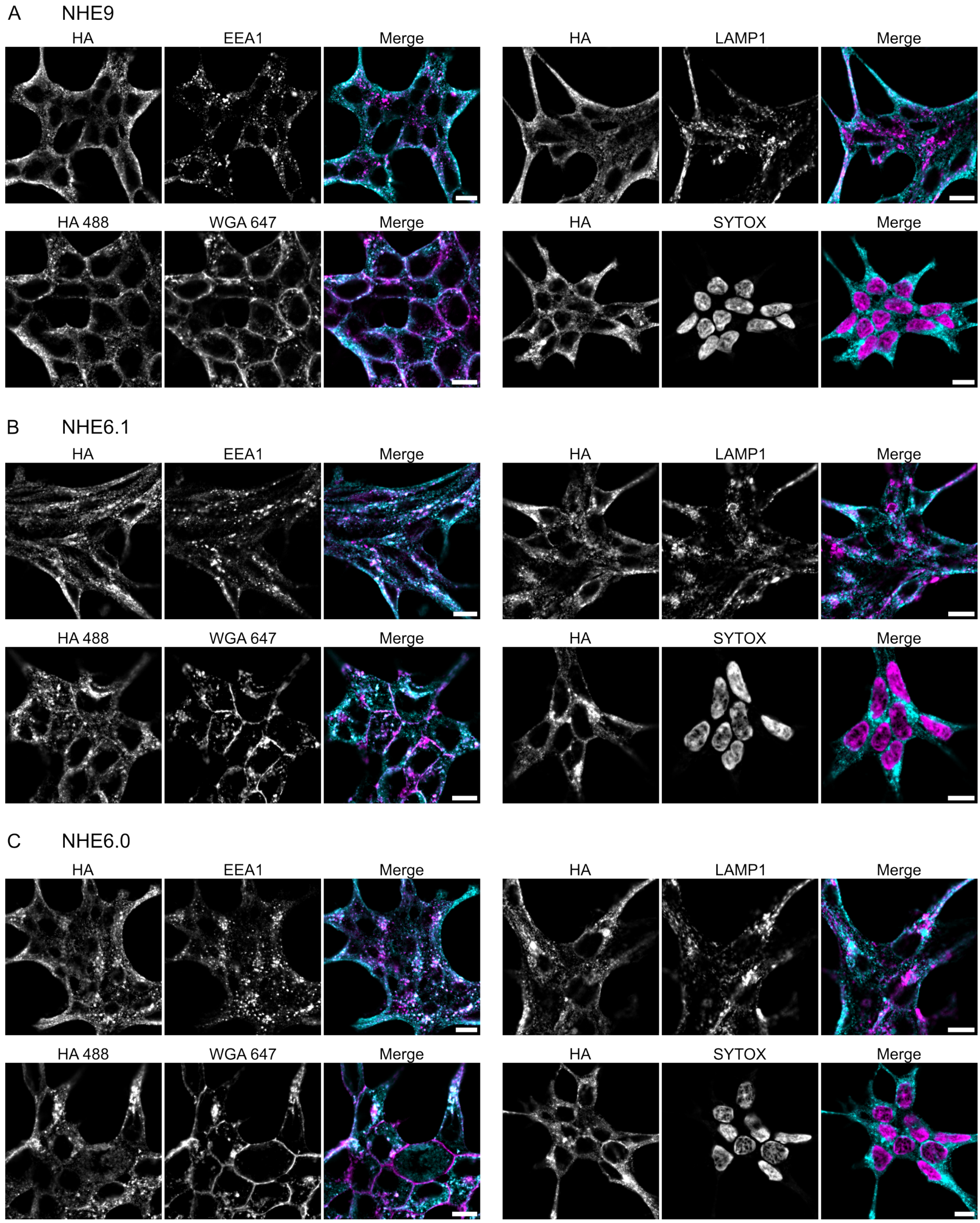

Supplementary Figure 1

Cellular distribution of NHE9, NHE6.1 and NHE6.0 in HEK cells. A. NHE9, **B.** NHE6.1 or **C.** NHE6.0 detected using anti-HA antibody against the HA-epitope tag of these proteins were immunofluorescently co-stained with organellar markers for early endosomes (EEA1), lysosomes (LAMP1), plasma membrane (WGA 647) and nuclei (SYTOX). Representative micrographs are shown of ≥10 micrographs acquired at 40× magnification for each condition. Scale bars are 10 µm.

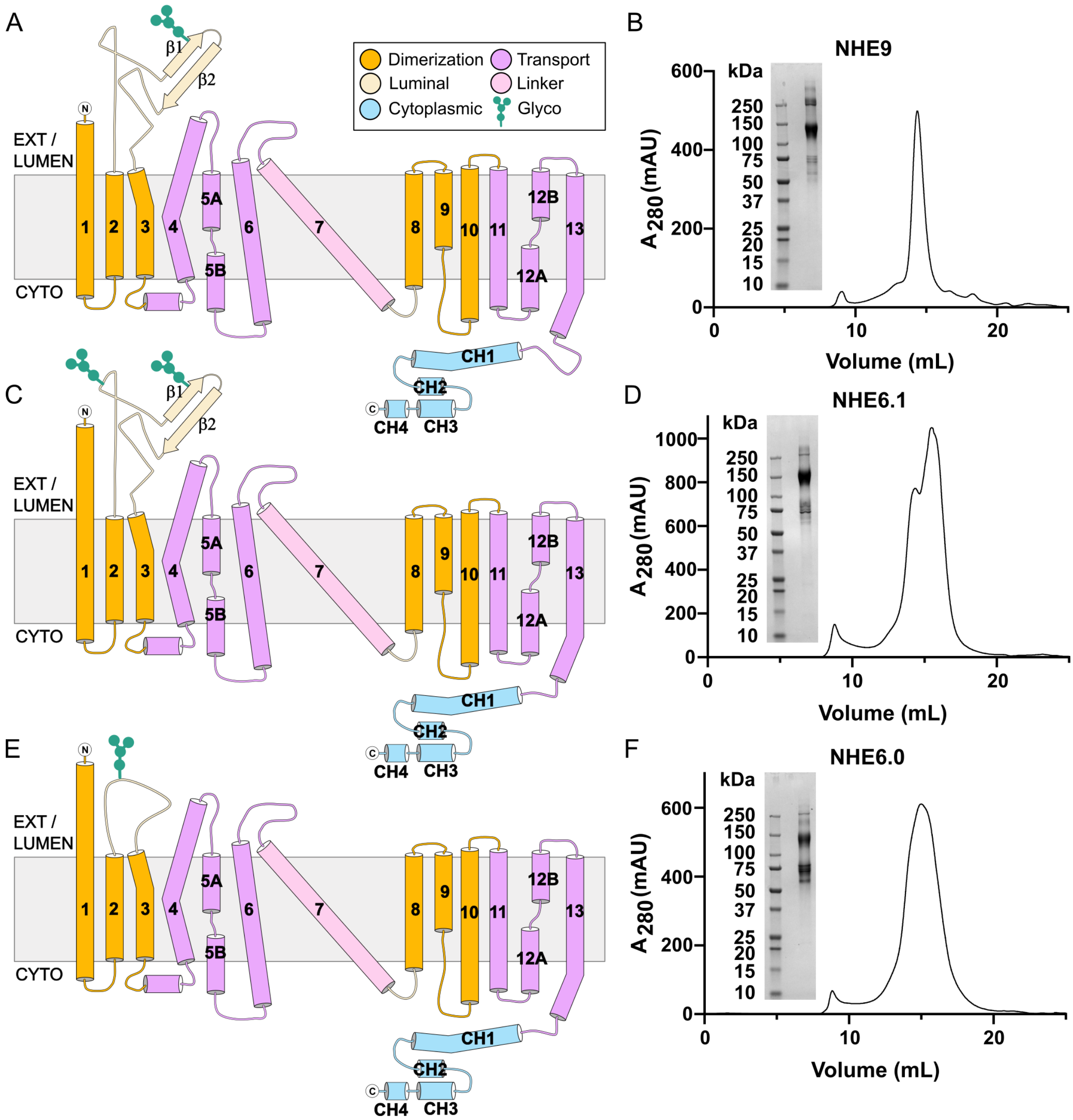

Supplementary Figure 2

Purification of NHE9 and two isoforms of NHE6. A. Topology map of NHE9, NHE6.1 (**C**) and NHE6.0 (**E**). Preparative size-exclusion chromatography of (**B**) NHE9, (**D**) NHE6.1 and (**F**) NHE6.0 on a Superose 6 Increase 10/300 GL column. Elution profiles were monitored by UV absorbance at 280 nm (A_{280}) and plotted in milli-absorbance units (mAU). Insets show SDS-PAGE analysis of the final concentrated protein samples obtained from the main peak fractions of each protein. Source data are provided as a Source Data file.

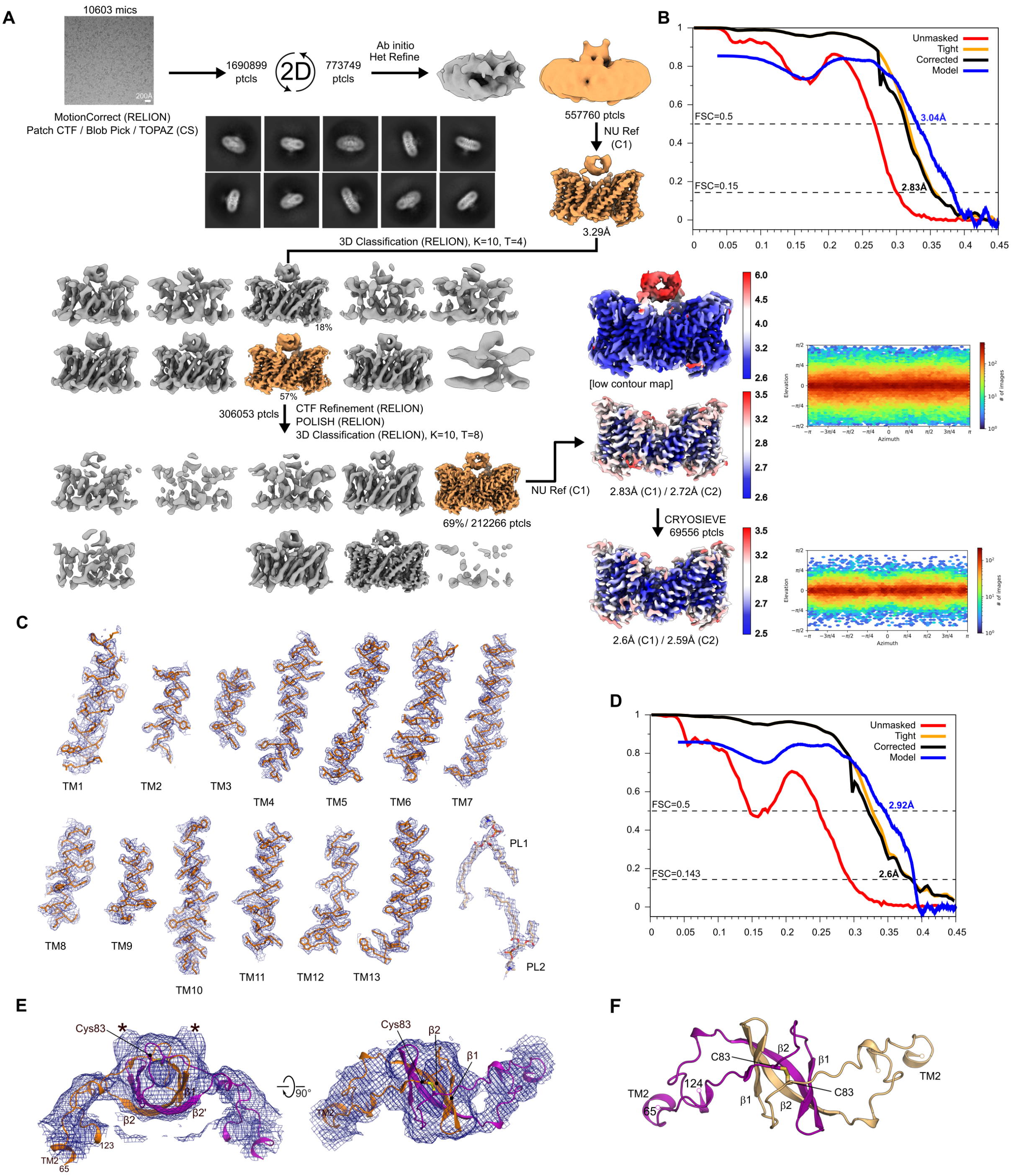

Supplementary Figure 3

Cryo-EM processing workflow and map quality for NHE9. A. Cryo-EM workflow to obtain final maps. Local resolution estimation was carried out in cryoSPARC using FSC filtering thresholding of 0.5. Final volumes are coloured by local resolution with a blue-white-red gradient. **B.** Gold standard FSC and map-to-model (blue) curves for reconstruction with all selected particles for full-length structure. **C.** Representative map quality for final core TM structure. The sharpened Coulomb potential map is shown superposed on the final model coordinates for each TM element and modelled lipids. **D.** Gold standard FSC and map-to-model (blue) curves for reconstruction with reduced subset of particles from CRYOSIEVE used for core TM modelling. **E.** Fit of AlphaFold2 TM2/3 luminal 'crown' model in blurred experimental map (blurring B-factor applied of 40Å^2). Asterisks highlight potential glycosylation of N96 in the crown domain. **F**. Ribbon structure of the crown domain (residues 65-124).

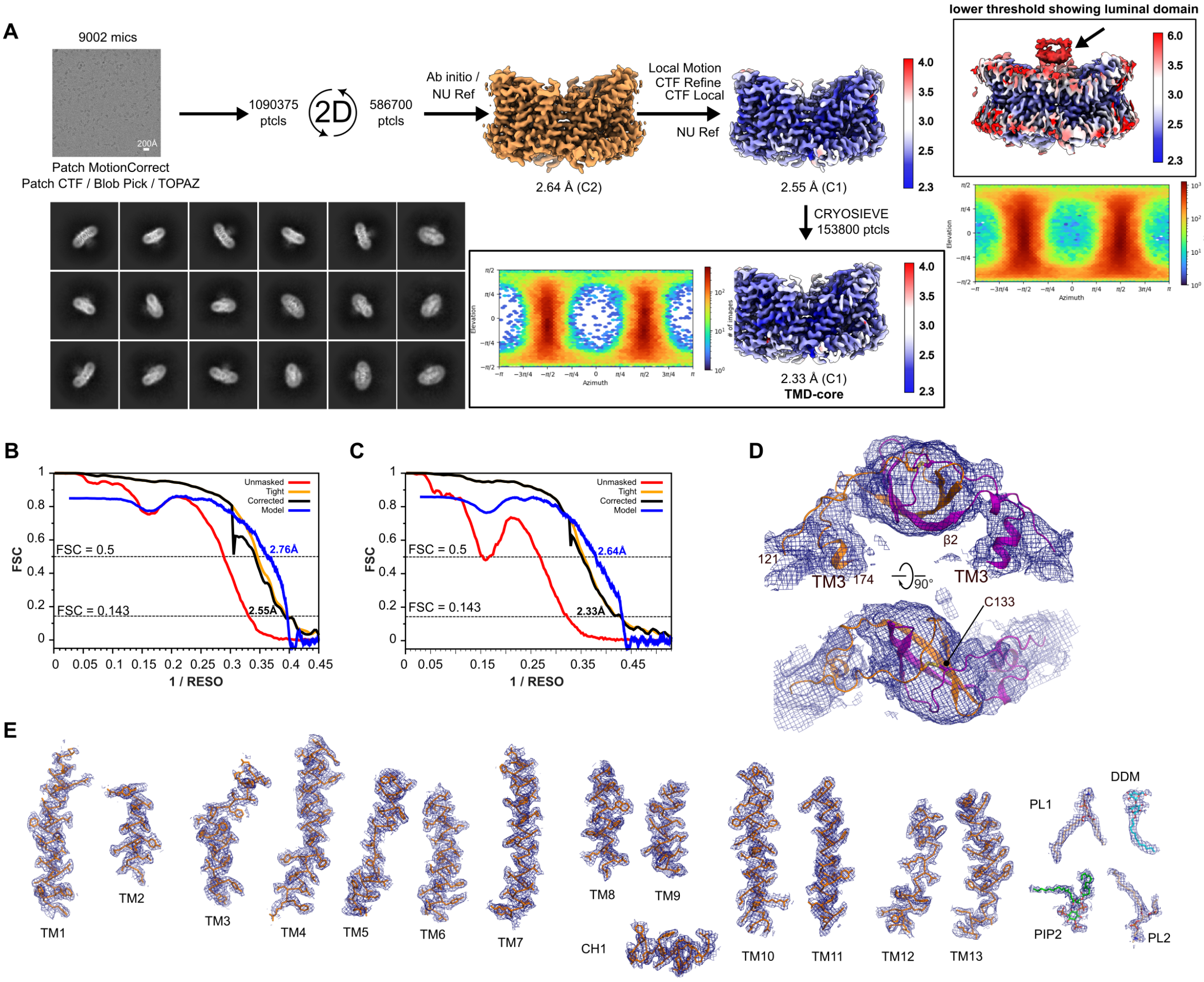

Supplementary Figure 4

Cryo-EM processing workflow and map quality for NHE6.1. A. Cryo-EM data workflow to obtain final maps. Local resolution estimation was carried out in cryoSPARC using FSC filtering thresholding of 0.5. **B.** Gold standard FSC and map-to-model (blue) curves for reconstruction with all polished particles used for full-length structure. **C.** Gold standard FSC and map-to-model (blue) curves for C1 reconstruction with reduced subset of particles from CRYOSIEVE used for core TM modelling. **D.** Luminal crown domain model, predicted by AlphaFold3, fitted to cryo-EM map obtained with pre-sieved particles and blurred with a *B*-factor of 40Å^2. **E.** Representative map quality for final core TM structure. The sharpened Coulomb potential map is shown superposed on the final model coordinates for each TM helix and modelled lipids.

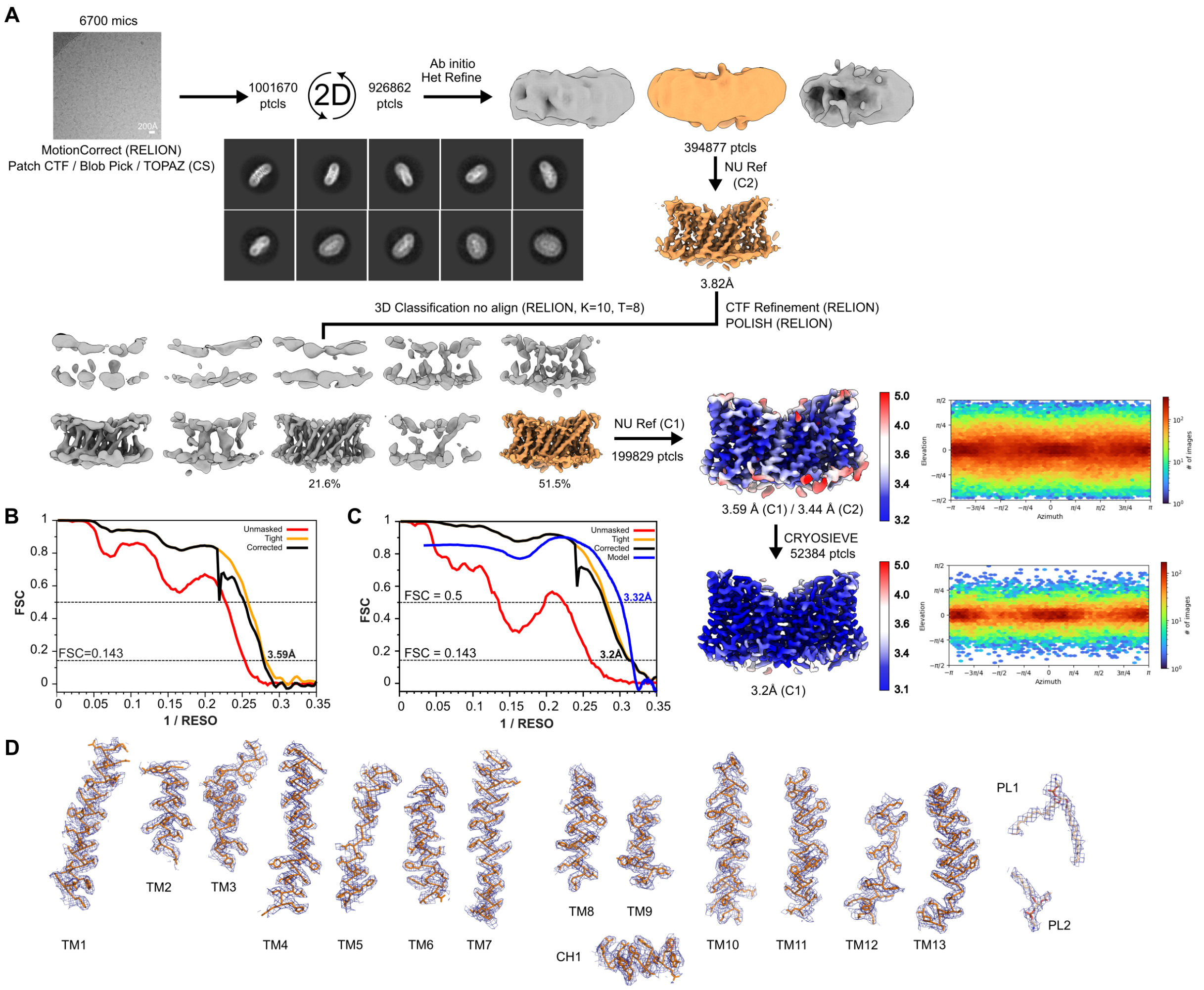

Supplementary Figure 5

Cryo-EM processing workflow and map quality for NHE6.0. A. Cryo-EM data workflow to obtain final maps. Local resolution estimation was carried out in cryoSPARC using FSC filtering thresholding of 0.5. **B.** Gold standard FSC curves for reconstruction with all polished particles prior to CRYOSIEVE. **C.** Gold standard FSC and map-to-model (blue) curves for final C1 reconstruction with reduced subset of particles from CRYOSIEVE used for model building. **D.** Representative map quality for final core TM structure. The sharpened Coulomb potential map is shown superposed on the final model coordinates for each TM helix and modelled lipids.

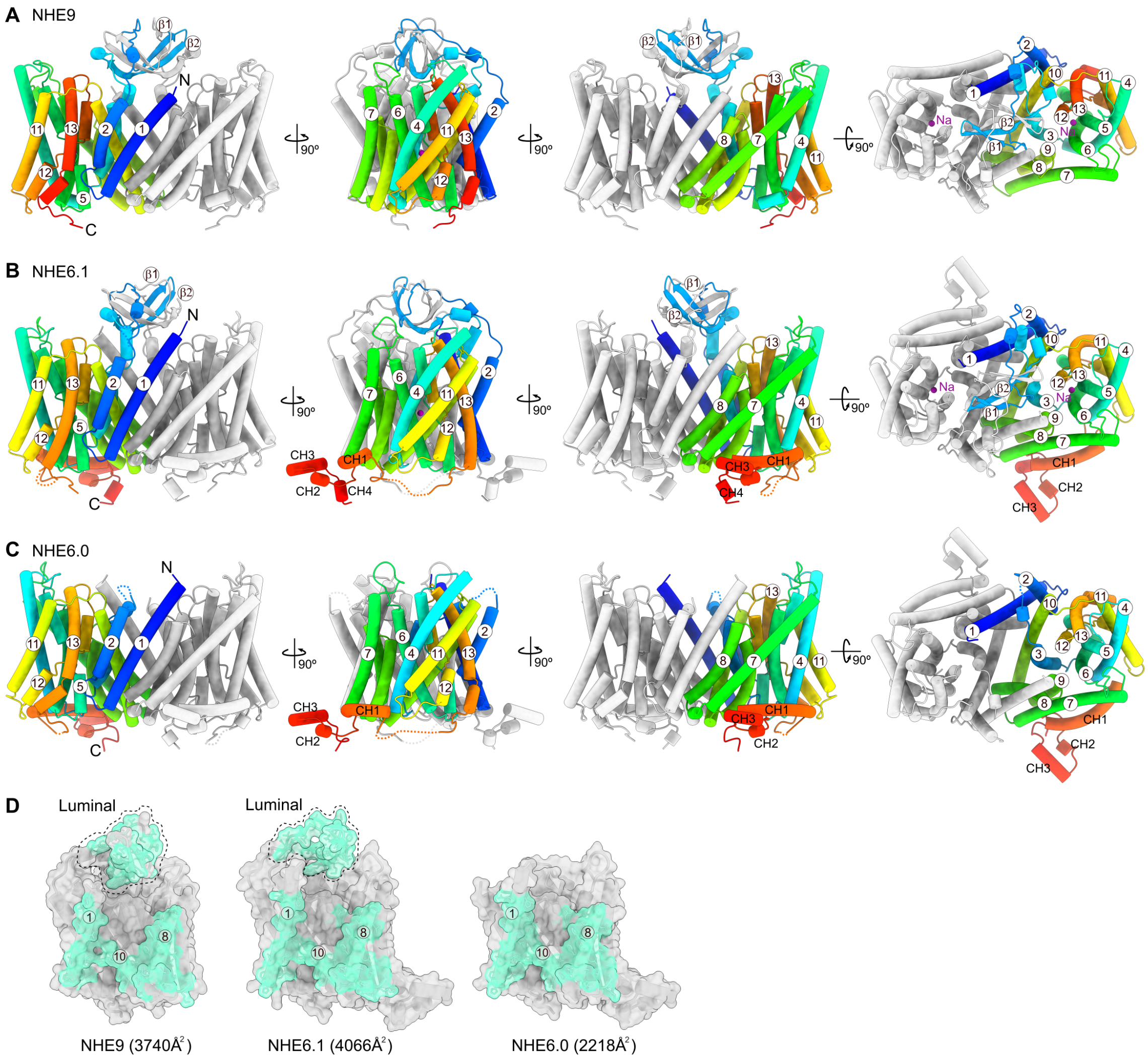

Supplementary Figure 6

Structures of NHE9, NHE6.1 and NHE6.0. Structures of **A.** NHE9, **B.** NHE6.1 and **C.** NHE6.0, coloured from the resolved N- to C-termini with a blue to red spectral gradient. **D.** Comparison of intermolecular dimer interfaces for NHE structures. Dimer interface is viewed looking from the adjacent monomer and residues within 4.2 Å are coloured aquamarine. Dimer interfacial area was calculated by PDBePISA.

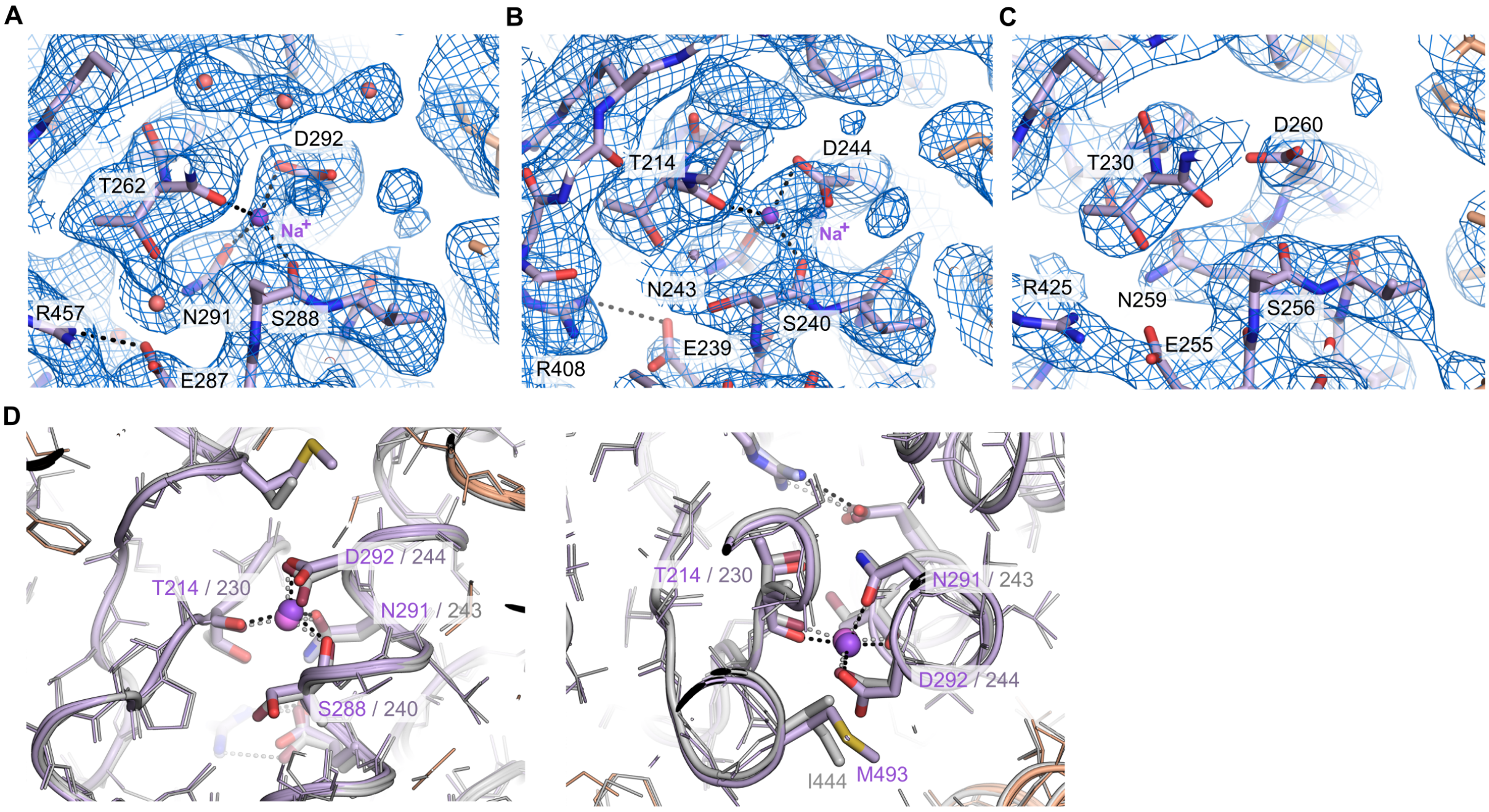

Supplementary Figure 7

Quality of Coulomb potential map. Cryo-EM density map for sodium binding site in (**A**) NHE6.1, (**B**) NHE9, (**C**) NHE6.0. Displayed cryo-EM maps were sharpened using the *B*-factor calculated by cryoSPARC after refinement, resampled to half the final pixel size and are displayed at a contour level just above that for the disordered detergent micelle. The resolution of the NHE6.0 map is not sufficient to resolve discrete Coulomb potential density for the sodium ion or solvent molecules. **D.** Comparison of sodium binding site for NHE6.1 (lilac) and NHE9 (grey).

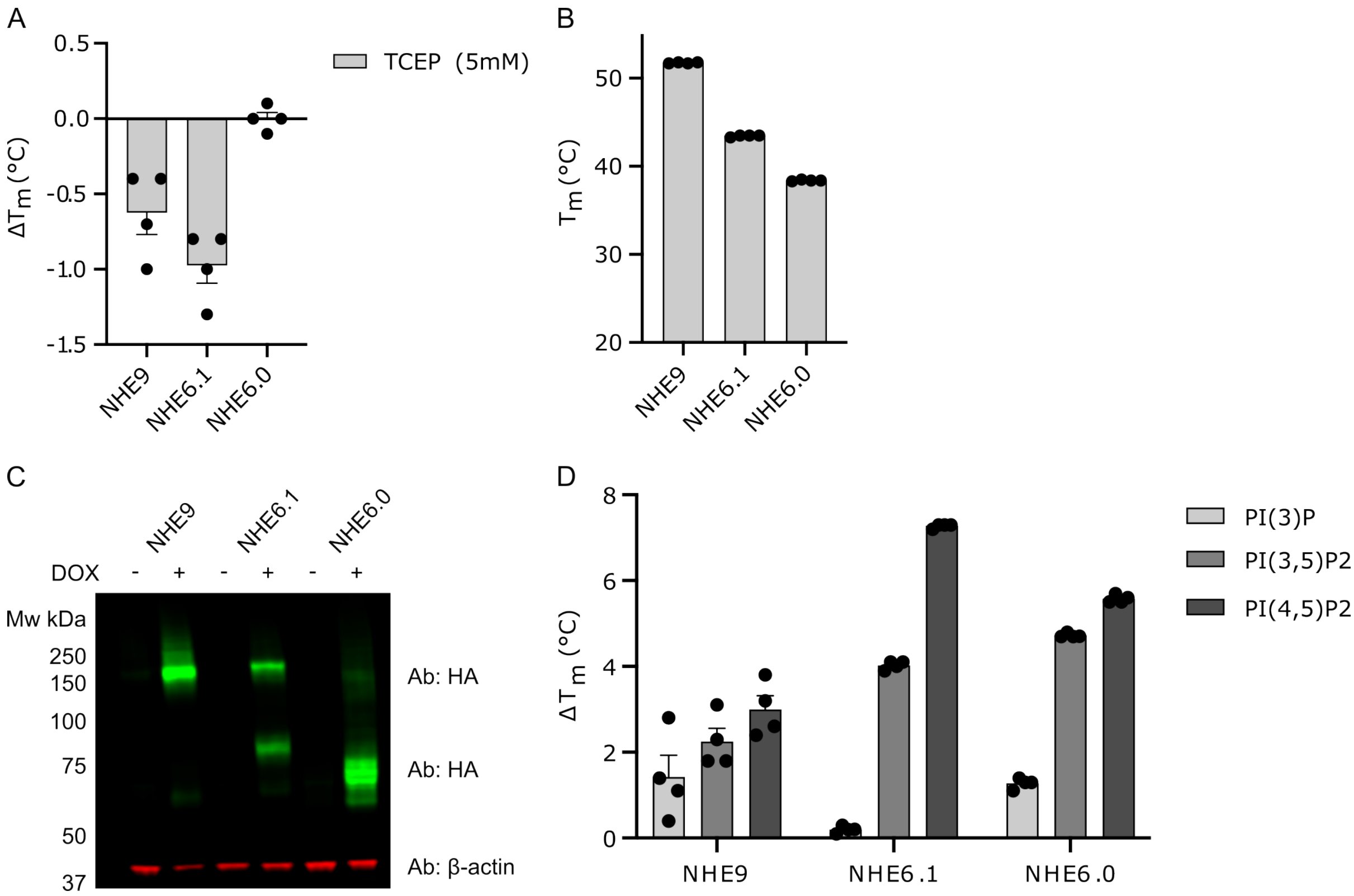

Supplementary Figure 8

Thermostability of NHE6 and NHE9. A. Thermal destabilization under reducing conditions. Plotted data shows the difference in thermostability without or with 5 mM TCEP added to purified NHE proteins. **B.** Thermostabilities of purified NHE proteins in SEC buffer. **C.** Western blot of overexpression cell lysates. The blot was probed with primary antibodies against the HA-epitope tag (NHE; green) and β-actin (loading control; red) and detected by fluorescently conjugated secondary antibodies using a LI-COR ODYSSEY CLx imager (see Methods). Representative Western blot results are shown (n=2 biological replicates). **D.** Thermostabilization of NHE6 and NHE9 with phosphoinositides. NanoDSF results are shown as mean ± SEM of n=4 technical replicates. Source data are provided as a Source Data file.

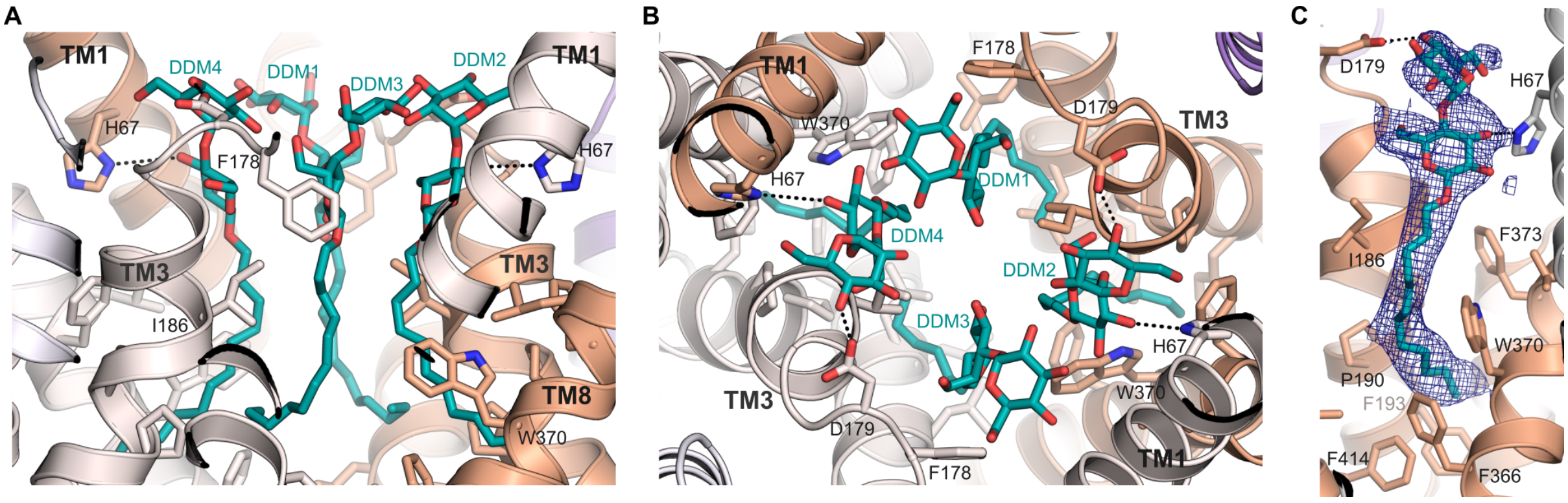

Supplementary Figure 9

Detergent molecules fill cavity at dimer interface. Structure of the dimer interface cavity in NHE6.1 on the luminal side of the transporter viewed (**A**) from membrane plane or (**B**) looking onto the luminal face. **C.** Coulomb potential density (final map sharpened with a *B*-factor of -25Å^2) for one of DDM molecules at the dimer interface.

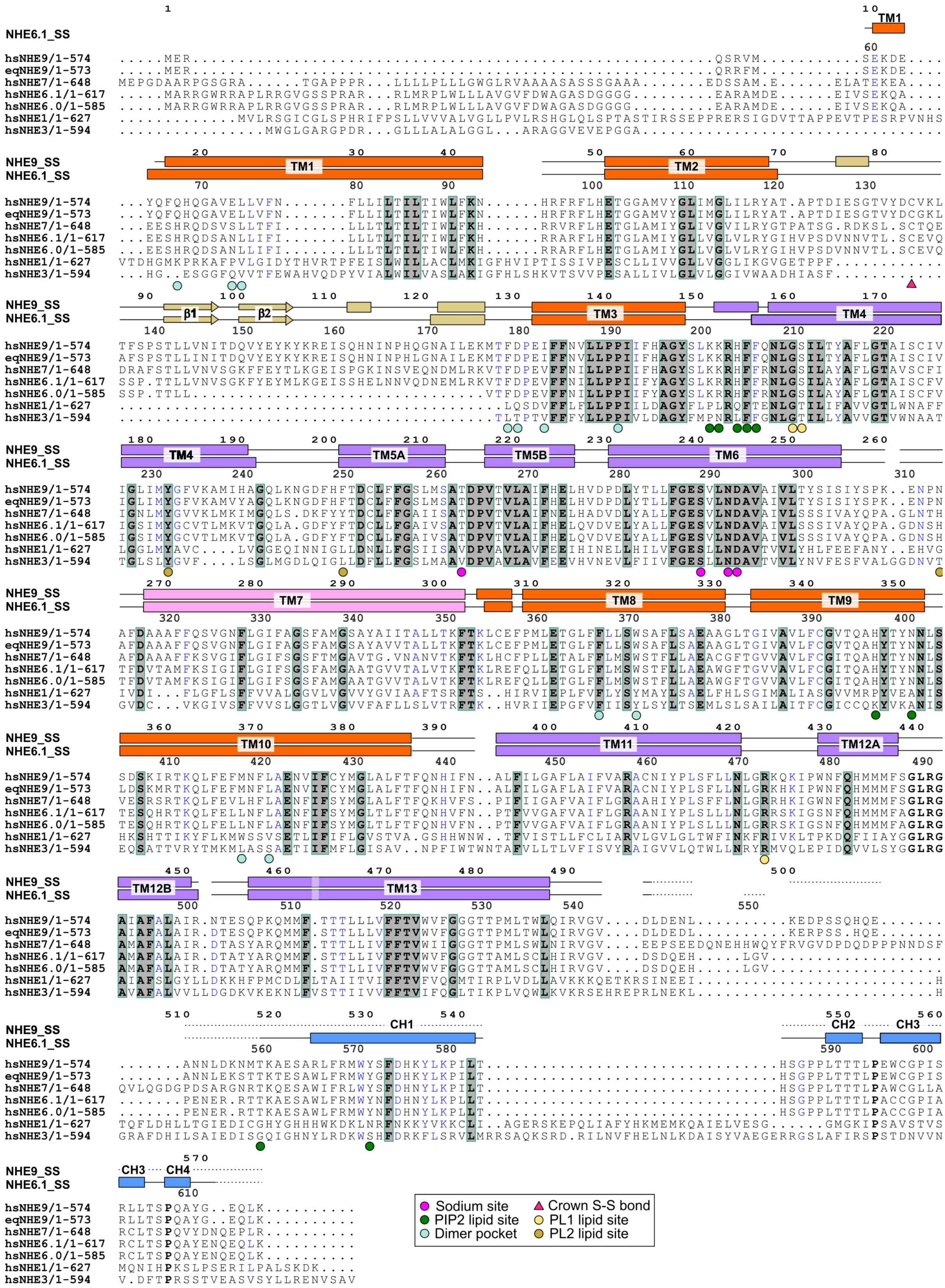

Supplementary Figure 10

Sequence alignment for NHE9, NHE6.1, NHE6.0 and related sequences. The secondary structure for NHE9 and NHE6.1 structures is shown above the alignment along with the respective sequence numbering for NHE9 (upper) and NHE6.1 (lower). The alignment is annotated indicating residues

involved in sodium and lipid binding. Sequences shown are human NHE9 (Q8IVBA), horse NHE9 (F7B113), human NHE7 (Q96T83), human NHE6.1 (Q92581-2), human NHE6.0 (Q92581-1), human NHE1 (P19634-1) and human NHE3 (P48764-1). For clarity C-terminal regions of all full-length sequences have been truncated as indicated by numbering.

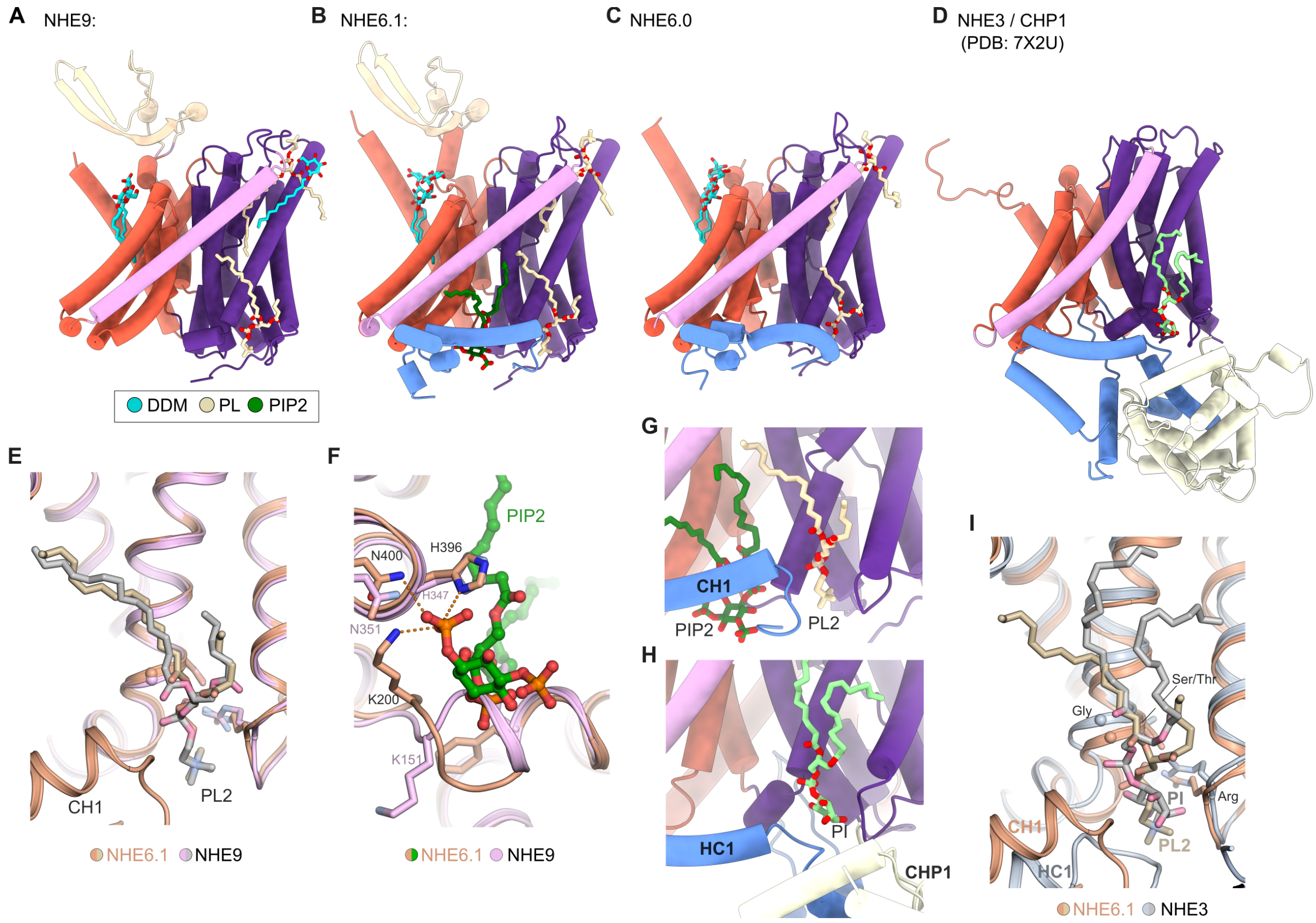

Supplementary Figure 11

Ordered lipid binding sites in NHEs. Structures of a single protomer from (**A**) NHE9, (**B**) NHE6.1, (**C**) NHE6.0, with resolved lipid sites highlighted. **D.** Overview of modelled lipid site in NHE3-CHP1 co-structure (PDB: 7X2U)[1]. **E**. Superposition of PL2 lipid site in NHE6.1 (pale orange) and NHE9 (pink/grey). **F**. Superposition of region in vicinity of PIP2 binding site observed in NHE6.1 (pale orange) with corresponding region in NHE9 structure (pink). The helical region between TM3/4 unwinds to accommodate PIP2. PL2 site in (**G**) NHE6.1 and (**H**) NHE3 (occupied by PI in PDB: 7X2U). **I.** Superposition NHE6.1 and NHE3 structures highlighting coincidence of modelled lipids. PL2 site is viewed as in (**G-H**) highlighting PL2 in NHE6.1 (pale orange/ yellow) and PI in NHE3 (grey).

Supplementary Table 1: Cryo-EM data collection, processing, refinement and validation statistics

	NHE6.1 (PIP2; core TM) EMD-53832 PDB 9R8N	NHE6.1 (PIP2;+luminal crown domain) EMD-53831 PDB 9R8M	NHE6.0 (apo) EMD-53829 PDB 9R8L	NHE9 (core TM) EMD-53828 PDB 9R8K	NHE9 (+ luminal crown domain) EMD-53827 PDB 9R8J
	**OPIC KRIOS F4i**		**eBIC KriosIII F4i**	**OPIC KRIOS F4i**	
**Data collection and processing**					
Magnification	130,000	130,000	130,000	165,000	165,000
Voltage (kV)	300	300	300	300	300
Electron exposure (e–/Å^2)	50	50	50	50	50
Defocus range (μm)	-0.8 to -2.2	-0.8 to -2.2	-1.0 to -2.4	-1.0 to -2.4	-1.0 to -2.4
Pixel size (Å)	0.932	0.932	0.921	0.7303	0.7303
Movies collected (curated)	9002(9002)	9002(9002)	6700 (6323)	11504 (10604)	11504 (10604)
Symmetry imposed	C1	C1	C1	C1	C1
Initial particle images	1090375	1090375	1001670	1690899	1690899
Final particle images	153800	586700	52384	69556	212266
Map resolution (Å)	2.33	2.55	3.20	2.60	2.83
FSC threshold	0.143	0.143	0.143	0.143	0.143
Map resolution range[1] (Å)	2.3 – 6.0	2.3 – 6.0	3.0 – 6.0	2.4 – 6.0	2.5 – 6.0
**Refinement**					
Initial model used (PDB code)	9R8L	9R8N / AF3	Alphafold2	Alphafold2	9R8K +AF2 multi
Model resolution[2] (Å)	2.64	2.76	3.32	2.90	3.02
FSC threshold	0.5	0.5	0.5	0.5	0.5
Map sharpening *B* factor (Å^2)	-52.3	+40	-66.8	-62.5	-25
Model composition					
Non-hydrogen atoms	8134	8838	7807	7096	7784
Protein residues	982	1078	969	864	960
Ligands	126	126	0	0	0
B factors (Å^2)					
Protein	51.72	132.35	64.16	62.53	124.66
Ligand	62.46	136.45	-	-	-
R.m.s. deviations					
Bond lengths (Å)	0.004	0.003	0.004	0.003	0.003
Bond angles (°)	0.639	0.560	0.566	0.535	0.489
Validation					
MolProbity score	1.20	1.30	1.32	1.25	1.34
Clashscore	3.70	3.34	3.51	2.80	3.60
Poor rotamers (%)	0.37	0.22	0.38	0.14	0.13
Ramachandran plot					
Favored (%)	97.84	97.01	96.97	96.96	96.86
Allowed (%)	2.16	2.99	3.03	3.04	3.14
Disallowed (%)	0	0	0	0	0

[1] Map resolution range is derived from local resolution estimation in cryoSPARC using the refinement mask and an FSC threshold of 0.5. The high resolution limit was determined by visually examining the unsharpened map coloured by local resolution in ChimeraX. The low resolution limit was chosen to reflect the poorly resolved protein features in the map.
[2] Model resolution as calculated by phenix.mtriage using the cryo-EM map used for refinement and the final refined model map (masked; FSC threshold of 0.5)

Supplementary References

1. Dong, Y., *et al.* Structural basis of autoinhibition of the human NHE3-CHP1 complex. *Sci Adv* **8**, eabn3925 https://doi.org/10.1126/sciadv.abn3925 (2022).
