# Supplementary material for: High-resolution structures of human NHE6 and NHE9 elucidate endosomal ion and lipid interactions
Source: Nat Commun. 2026 Sep 3;17:10478. doi: 10.1038/s41467-026-77254-0 (PMC13635234; doi:10.1038/s41467-026-77254-0)
Supplement: Supplementary file 2 [file 41467_2026_77254_MOESM2_ESM.pdf]

nature portfolio

Corresponding author(s): Kilian V.M. Huber

Last updated by author(s): Jul 20, 2026

Reporting Summary

Nature Portfolio wishes to improve the reproducibility of the work that we publish. This form provides structure for consistency and transparency in reporting. For further information on Nature Portfolio policies, see our Editorial Policies and the Editorial Policy Checklist.

Statistics

For all statistical analyses, confirm that the following items are present in the figure legend, table legend, main text, or Methods section.

n/a	Confirmed	
☐	☒	The exact sample size (*n*) for each experimental group/condition, given as a discrete number and unit of measurement
☐	☒	A statement on whether measurements were taken from distinct samples or whether the same sample was measured repeatedly
☒	☐	The statistical test(s) used AND whether they are one- or two-sided<br>*Only common tests should be described solely by name; describe more complex techniques in the Methods section.*
☒	☐	A description of all covariates tested
☒	☐	A description of any assumptions or corrections, such as tests of normality and adjustment for multiple comparisons
☐	☒	A full description of the statistical parameters including central tendency (e.g. means) or other basic estimates (e.g. regression coefficient) AND variation (e.g. standard deviation) or associated estimates of uncertainty (e.g. confidence intervals)
☒	☐	For null hypothesis testing, the test statistic (e.g. *F*, *t*, *r*) with confidence intervals, effect sizes, degrees of freedom and *P* value noted<br>*Give P values as exact values whenever suitable.*
☒	☐	For Bayesian analysis, information on the choice of priors and Markov chain Monte Carlo settings
☒	☐	For hierarchical and complex designs, identification of the appropriate level for tests and full reporting of outcomes
☒	☐	Estimates of effect sizes (e.g. Cohen's *d*, Pearson's *r*), indicating how they were calculated

Our web collection on statistics for biologists contains articles on many of the points above.

Software and code

Policy information about availability of computer code

Data collection	Cryo-EM data were collected with EPU (version 3.6.0.6389; Thermo Fisher Scientific).
Data analysis	Cryo-EM data were processed and analyzed with cryoSPARC (version 3.3.1; Structura Biotechnology). Additional data processing was performed with the following standard packages - TOPAZ (v0.2.4), PYEM (v 0.5), RELION (v4), CRYOSIEVE (v. 1.2.3), crYOLO (v1.9.6), Grade2 (v1.6.0). Structural models were built using Coot (v0.9.8.93) and ISOLDE (v1.3) with model refinement performed in Phenix (v1.21.1_5286). Molecular visualisation, analysis and figure generation were performed using PyMOL 2.2.0 (Schrodinger), ChimeraX v1.3 (UCSF Resource for Biocomputing, Visualization, and Informatics), Fiji (ImageJ v1.54p) and Prism 11.0.0 (GraphPad).

For manuscripts utilizing custom algorithms or software that are central to the research but not yet described in published literature, software must be made available to editors and reviewers. We strongly encourage code deposition in a community repository (e.g. GitHub). See the Nature Portfolio guidelines for submitting code & software for further information.

Data

Policy information about availability of data

All manuscripts must include a data availability statement. This statement should provide the following information, where applicable:

- Accession codes, unique identifiers, or web links for publicly available datasets
- A description of any restrictions on data availability
- For clinical datasets or third party data, please ensure that the statement adheres to our policy

The data that support this study are available from the corresponding authors upon request. The cryo-EM maps and models generated in this study have been

deposited in the Electron Microscopy Data Bank (EMDB) and the Protein Data Bank (PDB), respectively. NHE9 core (EMD-53828 [https://www.ebi.ac.uk/pdbe/entry/emdb/EMD-53828], PDB 9R8K [https://doi.org/10.2210/pdb9R8K/pdb]), NHE9 including luminal domain (EMD-53827 [https://www.ebi.ac.uk/pdbe/entry/emdb/EMD-53827], PDB 9R8J [https://doi.org/10.2210/pdb9R8J/pdb]), NHE6.0 (EMD-53829 [https://www.ebi.ac.uk/pdbe/entry/emdb/EMD-53829], PDB 9R8L [https://doi.org/10.2210/pdb9R8L/pdb]), NHE6.1 core / PIP2 complex (EMD-53832 [https://www.ebi.ac.uk/pdbe/entry/emdb/EMD-53832], PDB 9R8N [https://doi.org/10.2210/pdb9R8N/pdb]) , NHE6.1 including luminal domain / PIP2 complex, (EMD-53831 [https://www.ebi.ac.uk/pdbe/entry/emdb/EMD-53831],PDB 9R8M [https://doi.org/10.2210/pdb9R8M/pdb]). The horse NHE9 and human NHE1 and NHE3 used in this study for structural comparisons are available under accession codes 8PVR [https://doi.org/10.2210/pdb8PVR/pdb], 7DSV [https://doi.org/10.2210/pdb7DSV/pdb] and 7X2U [https://doi.org/10.2210/pdb7X2U/pdb] respectively. The protein detection, protein isolation and biophysical characterization data generated in this study are provided in the Supplementary Information/Source Data file. The source data underlying Supplementary Figure 2B,D,F and Supplementary Figure 8 are provided as a Source Data file.

Research involving human participants, their data, or biological material

Policy information about studies with human participants or human data. See also policy information about sex, gender (identity/presentation), and sexual orientation and race, ethnicity and racism.

Reporting on sex and gender	N/A
Reporting on race, ethnicity, or other socially relevant groupings	N/A
Population characteristics	N/A
Recruitment	N/A
Ethics oversight	N/A

Note that full information on the approval of the study protocol must also be provided in the manuscript.

Field-specific reporting

Please select the one below that is the best fit for your research. If you are not sure, read the appropriate sections before making your selection.

☒ Life sciences ☐ Behavioural & social sciences ☐ Ecological, evolutionary & environmental sciences

For a reference copy of the document with all sections, see nature.com/documents/nr-reporting-summary-flat.pdf

Life sciences study design

All studies must disclose on these points even when the disclosure is negative.

Sample size	CryoEM data collection sizes were chosen to ensure reproducibility based on prior experimental experience. No statistical methods were used to predetermine sample sizes as this manuscript does not contain data for which statistical analysis was required.
Data exclusions	No data was excluded during this study.
Replication	Cryo-EM data collection was carried out on a single sample in each case. Immunofluorescence was executed once and representative micrographs are shown of ≥10 micrographs acquired at 40× magnification for each condition. Western blotting was carried out with two biological replicates. For protein thermostability measurements (nanoDSF), TCEP was run with four technical replicates and all other experiments were conducted twice in four technical replicates. All replicates were successful.
Randomization	For cryo-EM studies, particles were randomly assigned to half-maps for resolution determination. No randomization was performed for binding assays as the study involved controlled in vitro experiments with predefined treatment conditions (e.g., adding defined reagents like lipid to recombinant proteins), where random allocation is not applicable.
Blinding	Blinding was not used. It is not relevant as outcomes of the experiments are not affected by knowledge of the sample.

Reporting for specific materials, systems and methods

We require information from authors about some types of materials, experimental systems and methods used in many studies. Here, indicate whether each material, system or method listed is relevant to your study. If you are not sure if a list item applies to your research, read the appropriate section before selecting a response.

Materials & experimental systems

n/a	Involved in the study
☐	☒ Antibodies
☐	☒ Eukaryotic cell lines
☒	☐ Palaeontology and archaeology
☒	☐ Animals and other organisms
☒	☐ Clinical data
☒	☐ Dual use research of concern
☒	☐ Plants

Methods

n/a	Involved in the study
☒	☐ ChIP-seq
☒	☐ Flow cytometry
☒	☐ MRI-based neuroimaging

Antibodies

Antibodies used	Alexa Fluor (AF) 488-conjugated anti-HA.11 Epitope Tag [16B12] (Biolegend #901509), anti-HA.11 epitope Tag (BioLegend #902301), anti-EEA1 (Proteintech #68065-1), anti-LAMP-1 (DSHB Cat# h4a3, RRID:AB_2296838), anti-β-Actin (Sigma-Aldrich #A5441), AF647-labeled goat anti-mouse IgG (Invitrogen #A21236), AF488-labeled goat anti-rabbit IgG (Invitrogen #A11034), AF680-labeled goat anti-rabbit IgG (Invitrogen #A-21076), AF790-labeled goat anti-mouse IgG (Invitrogen #A11375).
Validation	AF488-conjugated anti-HA.11 Epitope Tag [16B12] (Biolegend #901509): Used at dilution 1:1000. Key applications: FC (quality tested), ICC (verified) (https://www.biolegend.com/en-us/products/alexa-fluor-488-anti-ha-11-epitope-tag-antibody-10993).<br>Anti-HA.11 epitope Tag (BioLegend #902301): Used at dilution 1:500 (IF) or 1:1000 (WB). Key applications: WB (quality tested), IP (verified), ICC (reported in literature) (https://www.biolegend.com/en-us/products/purified-anti-ha11-epitope-tag-antibody-11375)<br>Anti-EEA1 (Proteintech #68065-1): Used at dilution 1:1000. Tested applications: WB, IHC, IF/ICC, ELISA (https://www.ptglab.com/products/EEA1-Antibody-68065-1-Ig.htm).<br>Anti-LAMP-1 (DSHB Cat# h4a3, RRID:AB_2296838): Used at dilution 1:200. Recommended applications: IF, IHC, WB (https://dshb.biology.uiowa.edu/H4A3?quantity=1).<br>Anti-β-Actin (Sigma-Aldrich #A5441): Used at dilution 1:500. Recommended applications: ELISA, IF, IHC, WB (https://www.sigmaaldrich.com/GB/en/product/sigma/a5441).<br>AF647-labeled goat anti-mouse IgG (Invitrogen #A21236): Used at dilution 1:800. Applications: IHC, ICC/IF, FC (https://www.thermofisher.com/antibody/product/Goat-anti-Mouse-IgG-H-L-Highly-Cross-Adsorbed-Secondary-Antibody-Polyclonal/A-21236).<br>AF488-labeled goat anti-rabbit IgG (Invitrogen #A11034): Used at dilution 1:800. Applications: ICC/IF, FC (https://www.thermofisher.com/antibody/product/Goat-anti-Rabbit-IgG-H-L-Highly-Cross-Adsorbed-Secondary-Antibody-Polyclonal/A-11034.<br>AF680-labeled goat anti-rabbit IgG (Invitrogen #A-21076):Used at dilution 1:5000. Applications: WB, ICC/IF (https://www.thermofisher.com/antibody/product/Goat-anti-Rabbit-IgG-H-L-Cross-Adsorbed-Secondary-Antibody-Polyclonal/A-21076).<br>AF790-labeled goat anti-mouse IgG (Invitrogen #A11375): Used at dilution 1:5000. Applications: WB, ICC/IF, FC (https://www.thermofisher.com/antibody/product/Goat-anti-Mouse-IgG-H-L-Cross-Adsorbed-Secondary-Antibody-Polyclonal/A11375).

Eukaryotic cell lines

Policy information about cell lines and Sex and Gender in Research

Cell line source(s)	Jump-In™ T-REx™ HEK293 (HEK293JI) Cells (Life Technologies through Fisher Scientific Austria, Order No.: K1889 (part of Kit No.:A15008).
Authentication	Parental Jump-In™ T-REx™ HEK293 cells were authenticated by STR profiling. The inducible NHE6.0, NHE6.1 and NHE9 cell lines were generated from this authenticated parental line. Expression of the codon-optimized transgenes was verified by RNA-seq., Western blotting and immunofluorescence imaging. Plasmid constructs were verified by Sanger sequencing prior to cell line generation. No additional STR profiling or karyotyping was performed on the derived stable cell lines.
Mycoplasma contamination	All cell lines were negative for mycoplasma.
Commonly misidentified lines<br>(See ICLAC register)	No commonly misidentified cell lines were used in this study.

Plants

Seed stocks	N/A
Novel plant genotypes	N/A
Authentication	N/A
