# Supplementary material for: High-resolution structures of human NHE6 and NHE9 elucidate endosomal ion and lipid interactions
Source: Nat Commun. 2026 Sep 3;17:10478. doi: 10.1038/s41467-026-77254-0 (PMC13635234; doi:10.1038/s41467-026-77254-0)
Supplement: Supplementary file 3 [file 41467_2026_77254_MOESM3_ESM.pdf]

nature portfolio

Peer Review File

High-resolution structures of human NHE6 and NHE9 elucidate endosomal ion and lipid interactions

Corresponding Author: Professor Kilian Huber

This file contains all reviewer reports in order by version, followed by all author rebuttals in order by version.

Version 0:

Reviewer comments:

Reviewer #1

(Remarks to the Author)
In the manuscript by Hansen et al, the authors solved the cryo-EM structures of human endosomal Na+/H+ exchangers NHE9 (2.6 Å) and two splice isoforms of NHE6 (NHE6.1 at 2.33 Å; NHE6.0 at 3.2 Å), all captured in inward-facing conformations. Thanks to the high-resolution obtained, the authors are able to describe the Na+ density in the canonical ion-binding site and identify multiple lipid densities, including two conserved phospholipid sites and one PIP2 site present in NHE6 but not in NHE9. They further provide a comparison between NHE6 splice isoforms that differ in the luminal "crown" insertion and dimer stability and propose a structural basis for RACK1 interaction.
Overall, I want to compliment the authors for a very well-written and well-presented manuscript and the high-resolution structures obtained, as such resolutions still remain challenging for membrane proteins.
Despite several closely related recent studies, the manuscript provides several new interesting insights. Yet it reads overall very structurally descriptive. While I am aware that these transporters can be difficult to assess functionally, the manuscript could benefit from more complementary biochemical assays and mutagenesis studies. As it is, several of the regulatory conclusions (lipid modulation, RACK1 interaction, isoform functional divergence), while convincing, are not yet strongly supported by functional validation. I also encourage the authors to expand the discussion section to place the findings more thoroughly in the context of the recent literature and to more explicitly compare their observations with these related studies.

- The authors interpret the non-protein density seen in the hydrophobic dimer interface pocket as DDM molecules. However, the structures were solved with the addition of lipids, and in a previous study of NHE9 (Kokane et al, 2025) using a similar strategy of purification with the addition of lipids yielded (PIP2)lipid densities in the cavity, to which a regulatory/functional role is attributed. Can the author discuss this further? Why were the detergent molecules not replaced by lipids? Do they assign a stronger functional role to the newly identified lipid sites?

- The authors describe two additional phospholipid sites present in their NHE6 and NHE9 structures. Do I assume it correctly that these pockets have not been described before? Or are all similarly described in the new BioRxiv paper from Feilen? Perhaps elaborate a bit more on this.

- The authors further describe an additional PIP2 binding site only present in NHE6.1 but not in NHE9. This is indeed a highly interesting new observation and of the major new insights provided by this work. Since no lipids were added to NHE6.0, do the authors assume the lipid density present here was endogenously purified or could it be PL too? Are mutations or any functional assays feasible within the scope of time that could further aid in characterizing this NHE6-specific site?

- Why is the proximal CTD region, encompassing CH1-CH4, crucial for the RACK1 interaction, not also visible in NHE9? In a previous study with horse NHE9 the region was resolved. The authors speculate that the region might be less stable in CTD due to lack of putative palmitoylation in NHE6. Are such densities for the plamitolyation visible? What if these sites are mutated? Here, some additional biochemistry or mutagenesis studies might provide valuable insights.

- Taking the new findings and those from related studies together, can the authors draw some more holistic mechanistic conclusions on the transport mechanism and its regulation? Lipid regulation, NHE6/NHE9 differences, etc.

Minor points:
- Page 4: it is disclosed that, despite the addition of an inhibitor, no density was observed in the structure. The authors could discuss this further here or in the discussion. I take it correctly that it was a NHE1 inhibitor? Why? Is there any information that it might also affect NHE9? If not this experiment could be another hint that the inhibitor does not bind to NHE9.
- Page 4 '… only residues Gln15-Asp494 were sufficiently resolved…' indicate Asp494 of how many
- Page 4: 'The overall NHE9 structure resembles that of the horse orthologue35, forming a homodimer in which each protomer adopts the NhaA-fold characteristic for this family of sodium-proton exchangers with well-defined transport and scaffold domains41 (monomer transmembrane (TM) core all atom RMSD 0.87 Å) (Fig. 1B).' does the rmsd refer to the both protomers or to the human NHE9 structure solved here and that of the horse NHE9 solved previously? RMSD or more detailed description on what is different or not might be helpful.
- Figure 3B: are the lipid binding pockets denoted by an asterisk the one being occupied and described in Figure 4? Perhaps mention this, e.g. that the shown molecular surface is only from the transporter, excluding the lipid density described later.
- Page 13: 'However, unlike simulations where sodium was mostly unable to bind protonated Asp244, our structures clearly show sodium binding to this residue, even at pH 7.5, where Asp is expected to be protonated. Therefore, while the typical pKa would predict this Asp is unprotonated, the local environment might enable protonation by altering this side chain's pKa47.' This paragraph is not entirely clear to me. Do you now expect that Asp244 is protonated in your structures? Based on what exactly and how calculated?
- Page 11: "To better resolve phosphoinositide-specific lipid binding sites, we supplemented purified NHE6.1 with the endosomally enriched PI(3,5)P2 during sample preparation. This regulatory lipid is clearly resolved in the cryo-EM map (Fig. 5A)." Also refer to Figure 4A to better guide the reader in the orientation/localisation of the binding site with respect to the transporter.
- Supplementary figure 2 could be extended to include a sequence alignment with respective numbering and label of relevant regions and residues for guidance when navigating between NHE6 and NHE9 (human and other recently published analogos structures)

Reviewer #2

(Remarks to the Author)
I co-reviewed this manuscript with one of the reviewers who provided the listed reports. This is part of the Nature Communications initiative to facilitate training in peer review and to provide appropriate recognition for Early Career Researchers who co-review manuscripts.

Reviewer #3

(Remarks to the Author)
The manuscript concerns elucidation of high-resolution cryo-EM structures of human endosomal Na+/H+ antiporters NHE9 and two isoforms of NHE6. Both transporters have significant physiological importance; therefore, more insight into their structure, regulation, and interactions with lipids is required to consider them as potential drug targets. The manuscript is very well written and comprehensible, the results are convincing and well documented, with corresponding images in the main text and the supplementary material.
The newly described high-resolution structures of human NHE9/NHE6 orthologs have not been resolved previously. In the case of NHE9, the structure is of higher resolution than previously obtained for the horse ortholog. In the case of NHE6, the parallel work of Feilen et al. 2025 submitted to BioRxiv is also mentioned in the manuscript and the resolution of their structure is also lower, which also indicates the significance of this work. The authors also present detailed NHE6 structures with bound lipids and show how variations in PIP and PIP2 levels may contribute to the thermostability of the partially overlapping yet non-redundant functional roles of NHE6 and NHE9 in different cellular compartments.
To improve the clarity and overall appeal of the results, I suggest the following corrections/revisions to the text and the addition/modification of some data. The individual points are listed in the order in which they appear in the manuscript.
Suggested corrections and modifications:
Abstract and Introduction
- Authors state in the abstract and in the introduction that both proteins act as electroneutral, but to my knowledge, only NHE9 has been mechanistically characterised as an electroneutral Na+/H+ exchanger; the stoichiometry of NHE6 has not been directly and quantitatively demonstrated. Therefore, I suggest rephrasing the sentence as: These proteins are widely considered as electroneutral exchangers ….
- Reference concerning NHE9 physiological functions should be more recent, e.g. https://doi.org/10.1111/ejn.70334 (in addition or instead of ref. No. 25)

Results and Discussion:
- Topology maps of NHE6.1 and NHE6.0 should be added to fig. S2, similarly to that for NHE9.
- Adding the figure with primary sequences (and their comparisons) with highlighted residues/regions described in the text to the supplementary material would improve the comprehensibility of the results and the information obtained about individual regions of the protein.
- Supplementary Fig. 8A – why the thermal stability is not shown the same way as in Suppl. Fig. 8D? Please revise the figure and the corresponding figure legend.

- Supplementary Fig. 8C (and corresponding text and methods) – the membrane shown looks as one picture. It is not clear if both antibodies were used at the same time? or if the membrane was reprobed, please clarify and adjust the corresponding picture of the membrane(s)
- Supplementary Fig. 10 – there is a typo in the last sentence NH6.1 – should be NHE6.1
- Is anything known under which conditions either of the isoforms NHE6.0 vs. NHE6.1 is expressed? These data can be included in the discussion to enhance the manuscript's physiological relevance. Is there any physiological explanation why NHE6.0 is monomeric, and NHE6.1 forms a dimer?
- The chapter Structural basis of RACK1 binding NHE6 – the results should be supported by a valid model of direct interaction between obtained structure of NHE6 and RACK1, which of the isoform is on Fig. 3?
- Resolved structures show sodium binding to the residue Asp244, even at pH 7.5, where Asp is expected to be protonated. How can this fact influence the stoichiometry of the transport? This should be discussed.
- What is known about the binding of inhibitors to the proteins studied? Could the authors support their concluding statement in the last paragraph of Discussion with a model of the interaction of NHE6/NHE9 with an inhibitor?

Version 1:

Reviewer comments:

Reviewer #1

(Remarks to the Author)
While I appreciate the authors’ responses to the reviewers’ comments, I would have welcomed a more comprehensive revision, particularly through the proposed broader discussion and additional mechanistic validation (e.g., targeted mutational analyses where feasible). Nevertheless, I acknowledge that the main findings remain of sufficient quality and interest, and given that the rebuttal letter will be published alongside the manuscript, I am satisfied that the authors have addressed the major concerns. I therefore support publication of the manuscript in its current form.

Reviewer #2

(Remarks to the Author)
I co-reviewed this manuscript with one of the reviewers who provided the listed reports. This is part of the Nature Communications initiative to facilitate training in peer review and to provide appropriate recognition for Early Career Researchers who co-review manuscripts.

Reviewer #3

(Remarks to the Author)
In the revised version of the manuscript, the authors have adequately addressed all of my comments.

Open Access This Peer Review File is licensed under a Creative Commons Attribution 4.0 International License, which permits use, sharing, adaptation, distribution and reproduction in any medium or format, as long as you give appropriate

credit to the original author(s) and the source, provide a link to the Creative Commons license, and indicate if changes were made.
In cases where reviewers are anonymous, credit should be given to 'Anonymous Referee' and the source.
The images or other third party material in this Peer Review File are included in the article's Creative Commons license, unless indicated otherwise in a credit line to the material. If material is not included in the article's Creative Commons license and your intended use is not permitted by statutory regulation or exceeds the permitted use, you will need to obtain permission directly from the copyright holder.
To view a copy of this license, visit https://creativecommons.org/licenses/by/4.0/

POINT-BY-POINT RESPONSE TO REVIEWERS

Reviewer 1:

Thanks to the high-resolution obtained, the authors are able to describe the Na^+ density in the canonical ion-binding site and identify multiple lipid densities, including two conserved phospholipid sites and one PIP2 site present in NHE6 but not in NHE9. They further provide a comparison between NHE6 splice isoforms that differ in the luminal "crown" insertion and dimer stability and propose a structural basis for RACK1 interaction. Overall, I want to compliment the authors for a very well-written and well-presented manuscript and the high-resolution structures obtained, as such resolutions still remain challenging for membrane proteins.

We thank the reviewer for their generous summary and constructive assessment.

1. *Despite several closely related recent studies, the manuscript provides several new interesting insights. Yet it reads overall very structurally descriptive. While I am aware that these transporters can be difficult to assess functionally, the manuscript could benefit from more complementary biochemical assays and mutagenesis studies. As it is, several of the regulatory conclusions (lipid modulation, RACK1 interaction, isoform functional divergence), while convincing, are not yet strongly supported by functional validation. I also encourage the authors to expand the discussion section to place the findings more thoroughly in the context of the recent literature and to more explicitly compare their observations with these related studies.*

We recognize that this work is primarily a high-resolution structural study of human NHE6 and NHE9, providing (i) direct visualization of the Na^+ site, (ii) comparative structures of two NHE6 splice isoforms, and (iii) defined lipid-binding sites including a phosphoinositide pocket in NHE6.

We appreciate the reviewer's acknowledgement of the technical complexity and difficulty in assessing organellar NHE functionally. We produced the NHE9 and NHE6 proteins used in this study from mammalian stable cell lines, making it nontrivial to produce mutants for functional studies. Given the technical constraints of robust functional reconstitution and technical limitations of assessing the possible regulatory or modulatory effects of lipids on this family of proteins, we have focused this study on high-resolution structural definition complemented by biophysical lipid-binding/stabilization readouts, and we have accordingly scoped our claims to ensure all mechanistic/regulatory statements are explicitly framed as structure-supported models rather than definitive functional conclusions.

Therefore, while the structural insights we present here open for exciting future research, follow-on studies will be needed (and are planned) to fully investigate these new hypotheses for NHE activity and regulation.

2. *The authors interpret the non-protein density seen in the hydrophobic dimer interface pocket as DDM molecules. However, the structures were solved with the addition of lipids, and in a previous study of NHE9 (Kokane et al, 2025) using a similar strategy of purification with the addition of lipids yielded (PIP2)lipid densities in the cavity, to which a regulatory/functional role is attributed. Can the author discuss this further? Why were the detergent molecules not replaced by lipids? Do they assign a stronger functional role to the newly identified lipid sites?*

We are grateful to the reviewer for raising this important point regarding our modeling of lipids and detergents within the structure. With our higher resolution, the features in the Coulomb potential map at the dimer interface are far more consistent with DDM than PIP2 in both shape and expected acyl chain length. Furthermore, we used DDM extraction for both NHE9 and NHE6 and the dimer interface features are broadly similar in all 3 structures regardless of whether PIP2 was supplemented. While we cannot exclude that these sites contain a very small fraction of PIP2, or other co-purified lipid, the cryo-EM maps strongly suggest that this pocket is occupied by detergent.

Nevertheless, in its native environment of the cell's endosomes this site is likely occupied by lipids, though the exact identity of the molecules may depend upon the composition of each leaflet of that membrane and will require detailed follow-up study. As PIP2 is primarily found in the cytoplasmic-facing leaflet, we expect NHE9 binding sites on this side of the membrane to be most relevant for the transporter's regulation.

Fundamentally, our observations are consistent with Kokane et al.'s observation of PIP2 co-purifying with the transporter. The lipid selectivity and regulatory role of each binding site will require further studies to consider differences in the NHE9 sequences between human and horse, experimental protocols, cryo-EM map quality and resolution, and the flip-flop of PIP2 and other lipids across the endosomal membrane to access each lipid binding site.

We have revised the manuscript to better detail our observations and their implications for NHE regulation, while also highlighting lipid regulation is likely to be a complex process and therefore require extensive and detailed future studies. The following paragraph has been included in the discussion:

"Hence, in addition to previously proposed roles of lipids to stabilise and regulate sodium-proton antiporters at the dimer interface, our results reveal PIP2 and other lipids binding NHE6 and NHE9 at the cytoplasmic face of the transport domain. However, this regulation

may be complex as the lipid composition will vary by organelle and signalling processes, and the effects of each lipid may differ across NHE genes and the differing species of origin used in experimental studies.”

3. *The authors describe two additional phospholipid sites present in their NHE6 and NHE9 structures. Do I assume it correctly that these pockets have not been described before? Or are all similarly described in the new BioRxiv paper from Feilen? Perhaps elaborate a bit more on this.*

We appreciate the reviewer’s comment and opportunity to clarify this: the two additional lipid sites, the PL sites we describe in our study, were not reported/discussed in Feilen et al. However, the PL2 site in our structures is like a previously modelled PI site in the NHE3-CHP1 co-structure (PDB: 7X2U), as shown in the revised Supplementary Figure 11.

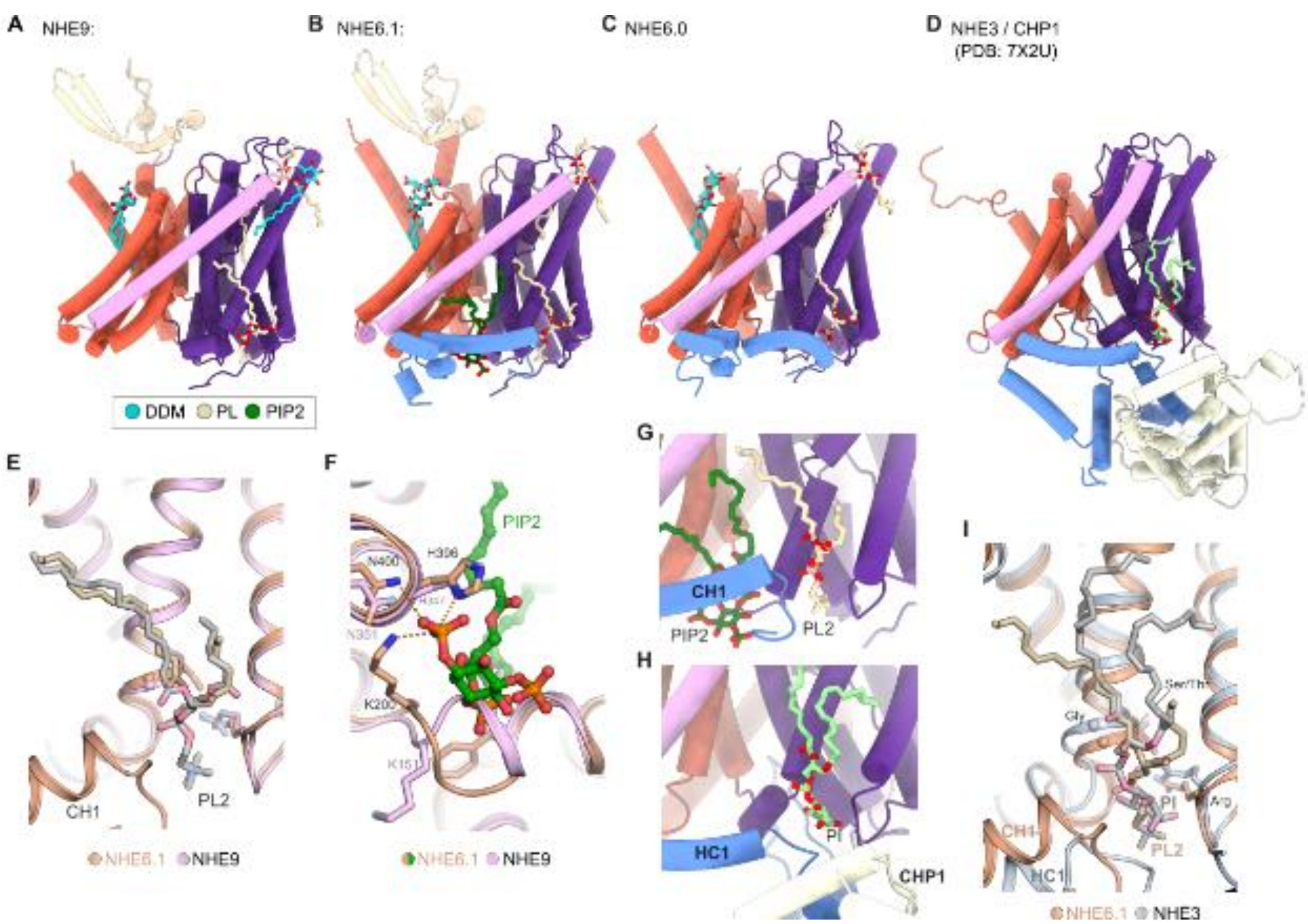

4. *The authors further describe an additional PIP2 binding site only present in NHE6.1 but not in NHE9. This is indeed a highly interesting new observation and of the major new insights provided by this work. Since no lipids were added to NHE6.0, do the authors assume the lipid density present here was endogenously purified or could it be PL too? Are mutations or any functional assays feasible*

within the scope of time that could further aid in characterizing this NHE6-specific site?

We thank the reviewer for highlighting this. NHE6.0 was prepared without exogenous phosphoinositide supplementation. The corresponding pocket shows weaker, partially ordered lipid-like density. Because headgroup features are insufficiently resolved, we interpret this density conservatively as an unassigned co-purified/endogenous lipid and have revised the Methods/Results to make this explicit:

The Results section is updated, reading:

"In the NHE6.0 structure prepared without PIP2 supplementation, this site adopts a similar conformation and appears to be partially occupied by an ordered, co-purifying lipid. However, the density is not sufficiently well resolved to allow definitive modelling."

The Methods section is updated, reading:

"The PL1/PL2 lipids were modelled as phosphatidylethanolamine based on the size and shape of the headgroup."

5. *Why is the proximal CTD region, encompassing CH1-CH4, crucial for the RACK1 interaction, not also visible in NHE9? In a previous study with horse NHE9 the region was resolved. The authors speculate that the region might be less stable in CTD due to lack of putative palmitoylation in NHE6. Are such densities for the palmitolyation visible? What if these sites are mutated? Here, some additional biochemistry or mutagenesis studies might provide valuable insights.*

We thank the reviewer for this important point. In our human NHE9 reconstruction, the proximal CTD segment encompassing CH1-CH4 is not resolved, whereas the analogous region is well defined in both NHE6 isoforms. First, the TM13-CH1 connecting loop, is eight residues longer in NHE9 versus NHE6. We hypothesize that this additional length increases flexibility/decoupling of the proximal CTD from the transmembrane core, reducing local order and thereby map visibility under our conditions. Second, differences in sample conditions likely contribute. Our human NHE9 structure was determined in DDM/CHS and resolve to residue 494, whereas the previously reported horse NHE9 structure (Winkelmann et al., 2020) was determined under different conditions (including LMNG-based detergent environments). Notably, even in that study, the resolved region encompassed only residues ~21–488 out of the protein's 645 amino acids, with only the CH1 helix modelled, indicating substantial CTD flexibility and partial disorder across preparations. Similarly, Feilen et al used different sample preparation for their NHE6 structure (GDN/CHS) and were only able to resolve the CH1 helix up to residue 585.

We have revised the manuscript to clarify that differences in this region between our human NHE6, NHE9, and the previous horse NHE9 most likely reflect both inherent differences between these NHE genes and experimental differences in local conformational heterogeneity and map resolvability:

“Notably, a structural feature that differs between NHE6 and NHE9 is the TM13-CH1 connecting loop, which is eight residues longer in NHE9. We hypothesize that this additional length increases flexibility, effectively decoupling the proximal CTD from the transmembrane core. This mobility likely reduces local order and thereby diminishes resolvability in the cryo-EM map under our sample conditions.”

Finally, we have reviewed the NHE6 maps for any density consistent with palmitoylation in the proximal CTD. We do not observe density that can be unambiguously attributed to lipid modification, which is not unexpected given detergent solubilization and the limitations of cryoEM for resolving flexible post-translational lipidations. We have revised the manuscript to refer to these sites as putative and based on software prediction to avoid over-interpretation:

“Moreover, three cysteine residues, including a predicted palmitoylation site”...

“We did not observe density compatible with confident modelling of a palmitoyl group in our NHE6 structures, though this is not surprising due to the detergent solubilized condition and the limitations of cryo-EM for resolving flexible post-translational lipidations. Nevertheless, it is tempting to speculate that the inability to resolve the RACK1-binding region in NHE9 is linked to stability differences in CTD regions, whereas NHE6 adopts a more stable configuration, possibly aided by palmitoylation, and hence allowing structure resolution.”

6. *Taking the new findings and those from related studies together, can the authors draw some more holistic mechanistic conclusions on the transport mechanism and its regulation? Lipid regulation, NHE6/NHE9 differences, etc.*

The reviewer raises an important point for understanding the transport and regulation of NHEs. There is increasing evidence that solute carrier proteins (CLC7s, NHEs, and SPNS2) are regulated by the second messenger PIP2. In addition to previously proposed roles of lipids to stabilize and regulate sodium-proton antiporters at the dimer interface, our results illustrate PIP2 and other lipids binding NHE6 and NHE9 at the cytoplasmic face of the transport domain. However, the lipid composition will vary by organelle and signaling processes, and the effects of each lipid may differ across NHE genes and the differing species of origin used in experimental studies. Therefore, while we expect our

results will contribute to a holistic understanding of NHE differences and regulation, achieving this goal will require focused studies of these and other factors. We have highlighted these issues in deciphering the full role of lipid regulation to NHEs in the revised discussion, as detailed above (please see response to 2).

Minor points:

7. *Page 4: it is disclosed that, despite the addition of an inhibitor, no density was observed in the structure. The authors could discuss this further here or in the discussion. I take it correctly that it was a NHE1 inhibitor? Why? Is there any information that it might also affect NHE9? If not this experiment could be another hint that the inhibitor does not bind to NHE9.*

We thank the reviewer for this comment. Their intuition is correct in our experimental design, process, and conclusions. We included the compound during grid preparation to assess whether it could stabilize the protein and improve conformational homogeneity based on the available understanding of the molecule's specificity at that time. However, no corresponding density was observed, and subsequent work suggests this chemotype is not broadly active across all NHE isoforms. Nevertheless, our cryo-EM dataset provided high-resolution maps of the proteins. We therefore focused our analysis on the protein structures and bound lipids, while noting the inhibitor for a complete description of our methods.

8. *Page 4 '… only residues Gln15-Asp494 were sufficiently resolved…' à indicate Asp494 of how many*

We agree with the reviewer, and we have revised the main text to reflect that we were able to sufficiently resolve Gln15-Asp494 of the 645 amino acids in NHE9:

"Although our construct used for protein purification was full-length (1-645), only residues Gln15-Asp494 were sufficiently resolved in the cryo-EM map for modelling."

9. *Page 4: 'The overall NHE9 structure resembles that of the horse orthologue35, forming a homodimer in which each protomer adopts the NhaA-fold characteristic for this family of sodium-proton exchangers with well-defined transport and scaffold domains41 (monomer transmembrane (TM) core all atom RMSD 0.87 Å) (Fig. 1B).' à does the rmsd refer to the both protomers or to the human NHE9 structure solved here and that of the horse NHE9 solved previously? RMSD or more detailed description on what is different or not might be helpful.*

We have clarified which regions of human and horse NHE9 protomers that the RMSD quoted refers to. We also briefly summarise the differences in the text:

“The core TM1-TM13 domain of human and horse NHE9 is structurally similar (all atom RMSD 0.87 Å) with only subtle differences observed in the conformation of the luminal loops and a shift in the positioning of the short helix between TM3 and TM4 (Fig. 1B).”

10. *Figure 3B: are the lipid binding pockets denoted by an asterisk the one being occupied and described in Figure 4? Perhaps mention this, e.g. that the shown molecular surface is only from the transporter, excluding the lipid density described later.*

We thank the reviewer for pointing this out. We have now revised the text and figure to clarify PIP2 and PL2 binding sites:

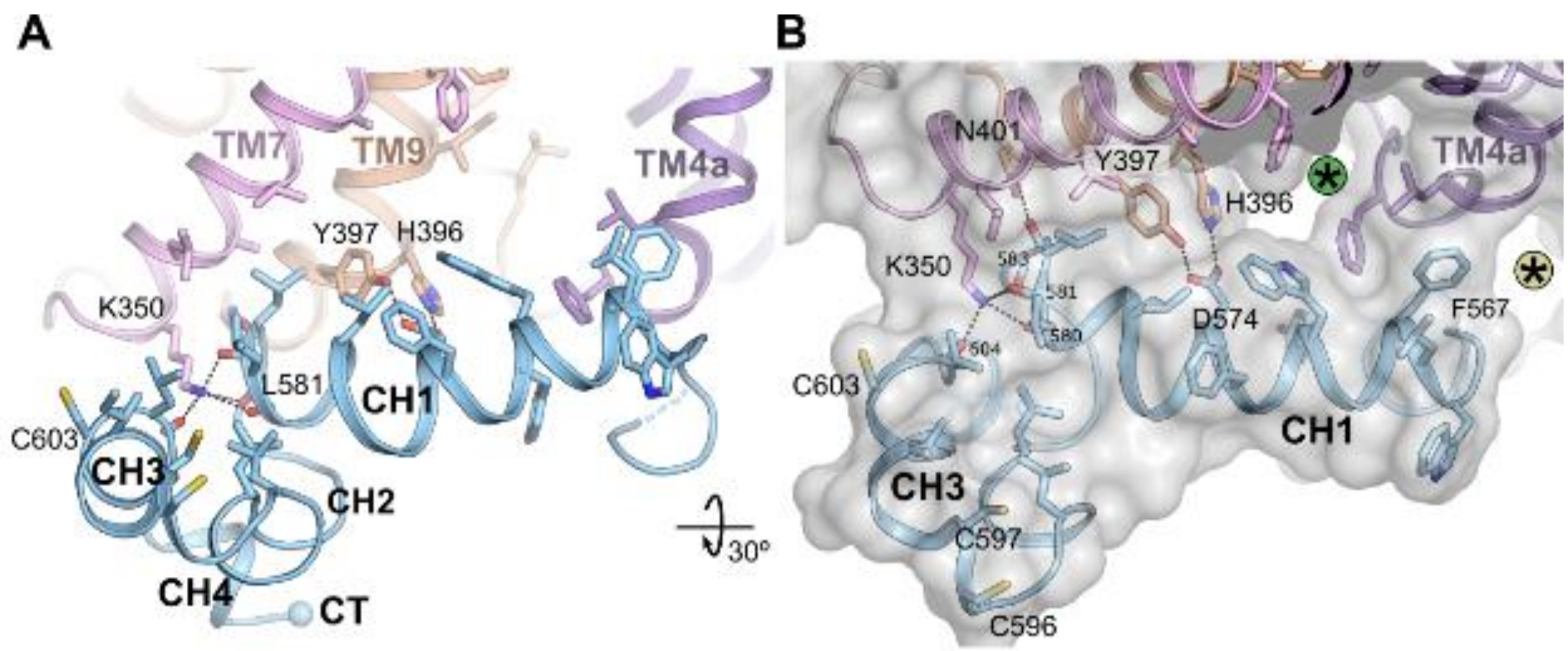

Figure legend now includes:

“The positions occupied by the PIP2 and PL2 lipids are indicated by coloured asterisks (green: PIP2; wheat: PL2).”

11. *Page 13: ‘However, unlike simulations where sodium was mostly unable to bind protonated Asp244, our structures clearly show sodium binding to this residue, even at pH 7.5, where Asp is expected to be protonated. Therefore, while the typical pKa would predict this Asp is unprotonated, the local environment might enable protonation by altering this side chain’s pKa47.’ à This paragraph is not entirely clear to me. Do you now expect that Asp244 is protonated in your structures? Based on what exactly and how calculated?*

The reviewer raises an important point. Cryo-EM at the present resolution does not directly report protonation states. Our inference that Asp244's protonation state is based on the bulk pKa for aspartate side chains and the transport mechanism of sodium-proton antiporters, Nevertheless, we have not directly determined the protonation state or pKa of this residue, and have more clearly stated this in the revised text:

"In the MD simulations, sodium was only able to bind NHE9 when the equivalent of Asp244 was unprotonated. This agrees with the expected protonation state of aspartate side chains at our experimental pH of 7.5, though Asp244's exact pKa may be modulated by the local environment[47] and cannot be directly determined from our cryo-EM maps."

12. *Page 11: "To better resolve phosphoinositide-specific lipid binding sites, we supplemented purified NHE6.1 with the endosomally enriched PI(3,5)P2 during sample preparation. This regulatory lipid is clearly resolved in the cryo-EM map (Fig. 5A)." - Also refer to Figure 4A to better guide the reader in the orientation/localisation of the binding site with respect to the transporter.*

We thank the reviewer for flagging this up and have changed the text accordingly.

13. *Supplementary figure 2 could be extended to include a sequence alignment with respective numbering and label of relevant regions and residues for guidance when navigating between NHE6 and NHE9 (human and other recently published analogos structures).*

The reviewer raises a valid point. In better guiding the audience, we have added a sequence alignment of relevant NHE genes. It has been included as Supplementary Figure 10:

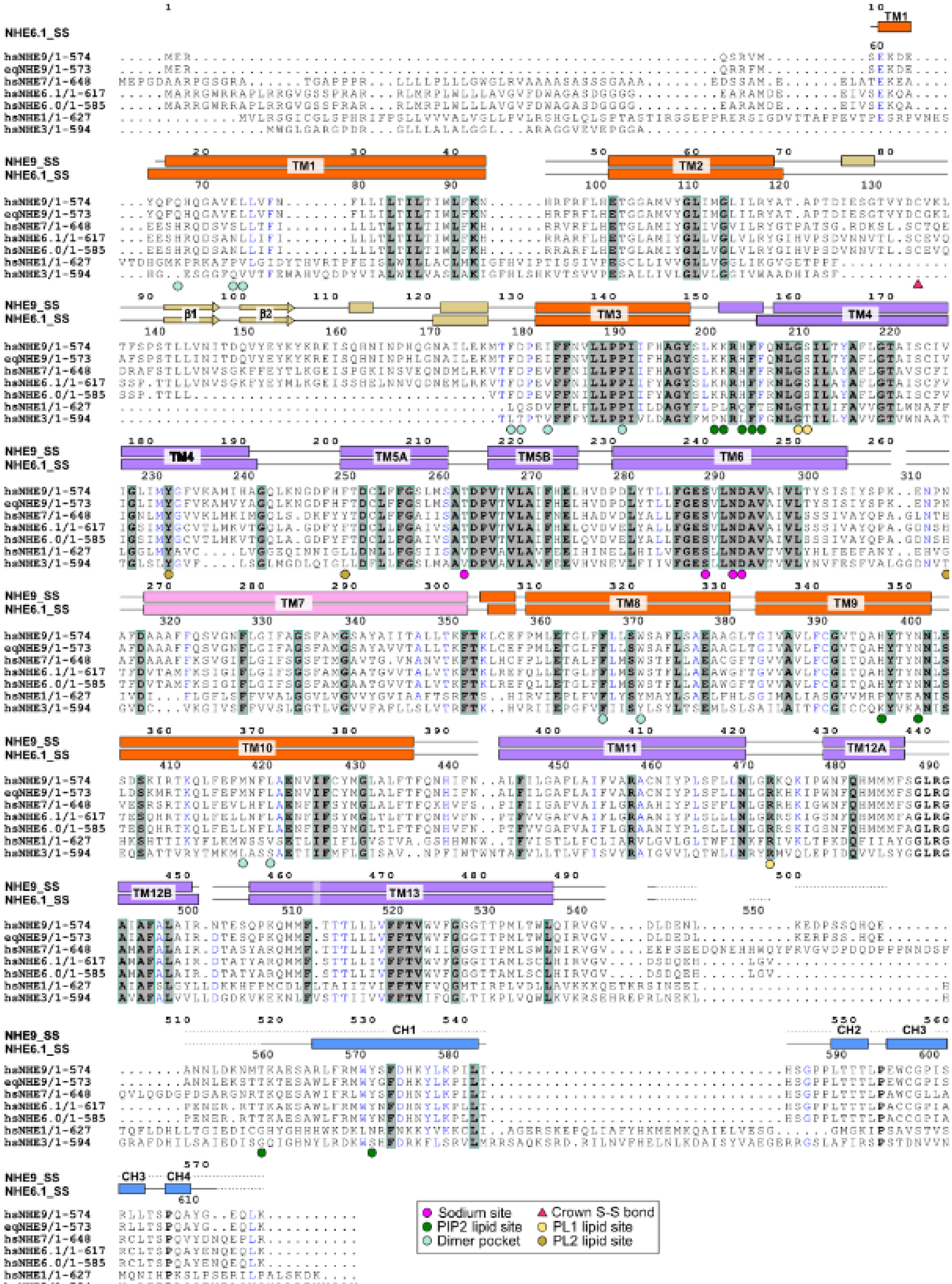

NHE6.1_SS
NHE9_SS
hsNHE9/1-574
eqNHE9/1-573
hsNHE7/1-648
hsNHE6.1/1-617
hsNHE6.0/1-585
hsNHE1/1-627
hsNHE3/1-594
TM1
TM2
β1
β2
TM3
TM4
TM5A
TM5B
TM6
TM7
TM8
TM9
TM10
TM11
TM12A
TM12B
TM13
CH1
CH2
CH3
CH4
Sodium site
Crown S-S bond
PIP2 lipid site
PL1 lipid site
Dimer pocket
PL2 lipid site

Reviewer 2:

I co-reviewed this manuscript with one of the reviewers who provided the listed reports. This is part of the Nature Communications initiative to facilitate training in peer review and to provide appropriate recognition for Early Career Researchers who co-review manuscripts.

We thank the reviewer for their contribution to the peer-review process and for their constructive feedback provided through the shared report.

Reviewer 3:

The manuscript is very well written and comprehensible, the results are convincing and well documented, with corresponding images in the main text and the supplementary material.

The newly described high-resolution structures of human NHE9/NHE6 orthologs have not been resolved previously. In the case of NHE9, the structure is of higher resolution than previously obtained for the horse ortholog. In the case of NHE6, the parallel work of Feilen et al. 2025 submitted to BioRxiv is also mentioned in the manuscript and the resolution of their structure is also lower, which also indicates the significance of this work. The authors also present detailed NHE6 structures with bound lipids and show how variations in PIP and PIP2 levels may contribute to the thermostability of the partially overlapping yet non-redundant functional roles of NHE6 and NHE9 in different cellular compartments.

We thank the reviewer for their positive comments and appreciation of our work's value to understanding NHE regulation.

1. *Authors state in the abstract and in the introduction that both proteins act as electroneutral, but to my knowledge, only NHE9 has been mechanistically characterised as an electroneutral Na+/H+ exchanger; the stoichiometry of NHE6 has not been directly and quantitatively demonstrated. Therefore, I suggest rephrasing the sentence as: These proteins are widely considered as electroneutral exchangers ….*

We thank the reviewer for pointing out that the stoichiometry of NHE6 transport hasn't yet been unequivocally determined experimentally, and that care needs to be taken in their description. We have amended the manuscript text as per the reviewer's suggestion:

"NHE6 and NHE9 are widely considered to function as electroneutral exchangers"...

2. *Reference concerning NHE9 physiological functions should be more recent, e.g. https://doi.org/10.1111/ejn.70334 (in addition or instead of ref. No. 25).*

We thank the reviewer for suggesting a more recent reference. We have replaced the previous reference (https://doi.org/10.3389/fncel.2014.00172) with the suggestion.

3. *Topology maps of NHE6.1 and NHE6.0 should be added to fig. S2, similarly to that for NHE9.*

We thank the reviewer for this suggestion and agree that these are valuable guides to understanding the architecture of these proteins. We have added these topology maps to Supplementary Figure 2:

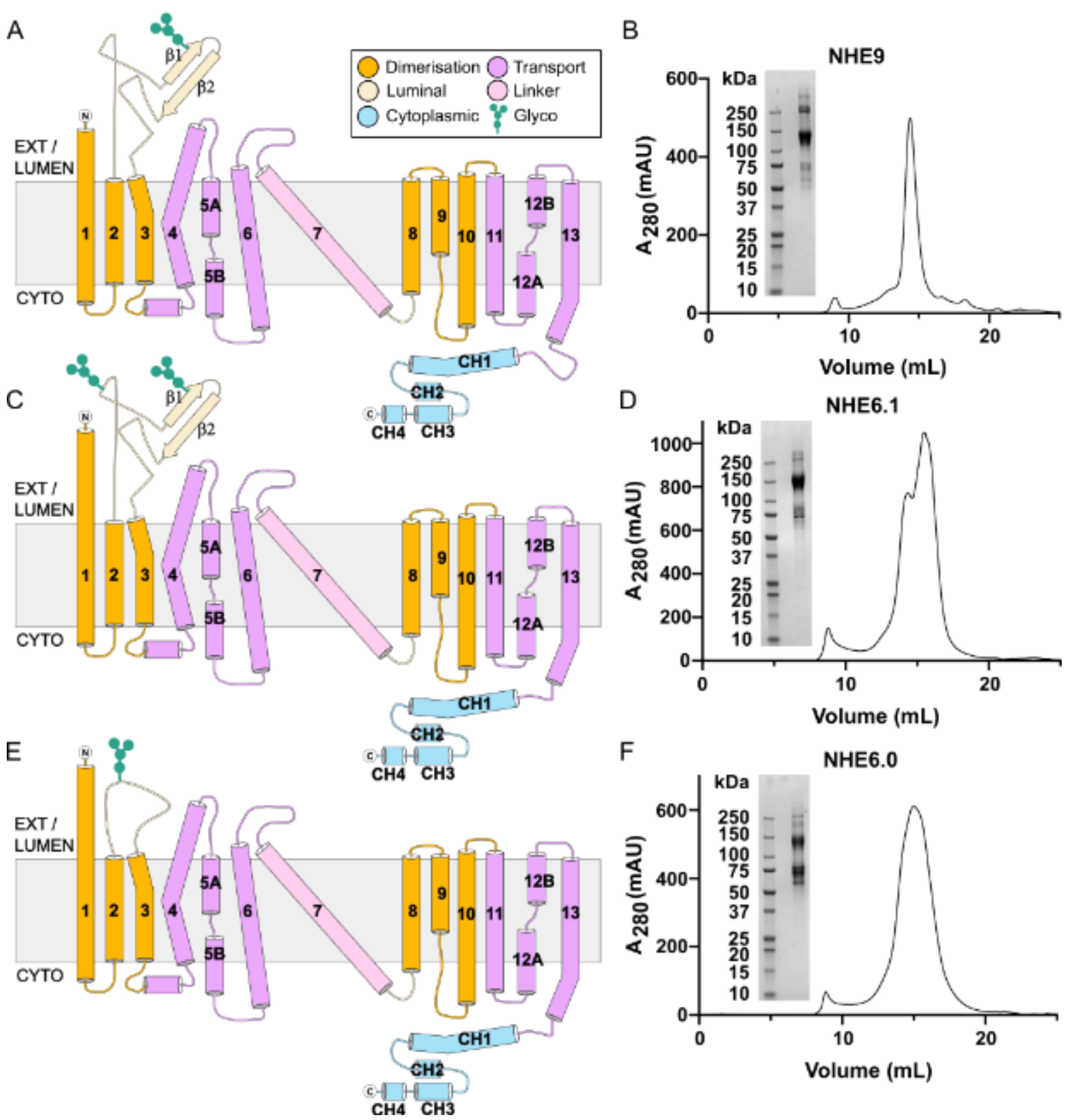

4. *Adding the figure with primary sequences (and their comparisons) with highlighted residues/regions described in the text to the supplementary material would*

improve the comprehensibility of the results and the information obtained about individual regions of the protein.

We thank the reviewer for this suggestion and have added a sequence alignment for relevant human and horse NHEs, indicating the key residues identified in our structural studies. This has been included in supplementary information as Supplementary Figure 10.

5. *Supplementary Fig. 8A – why the thermal stability is not shown the same way as in Suppl. Fig. 8D? Please revise the figure and the corresponding figure legend.*

The panels in Supplementary Figure 8 show each protein's thermal stabilization or destabilization in response to lipids and reducing conditions, respectively, and hence may appear different. We have revised the Supplementary Figure panel 8A / D to more clearly illustrate this.

6. *Supplementary Fig. 8C (and corresponding text and methods) – the membrane shown looks as one picture. It is not clear if both antibodies were used at the same time? or if the membrane was reprobed, please clarify and adjust the corresponding picture of the membrane(s).*

We thank the reviewer for this suggestion. We now show the western blot with the fluorescent green and red channel colors on a black background. We have also amended the figure legend and methods to clarify this. Revised Supplementary Figure 8:

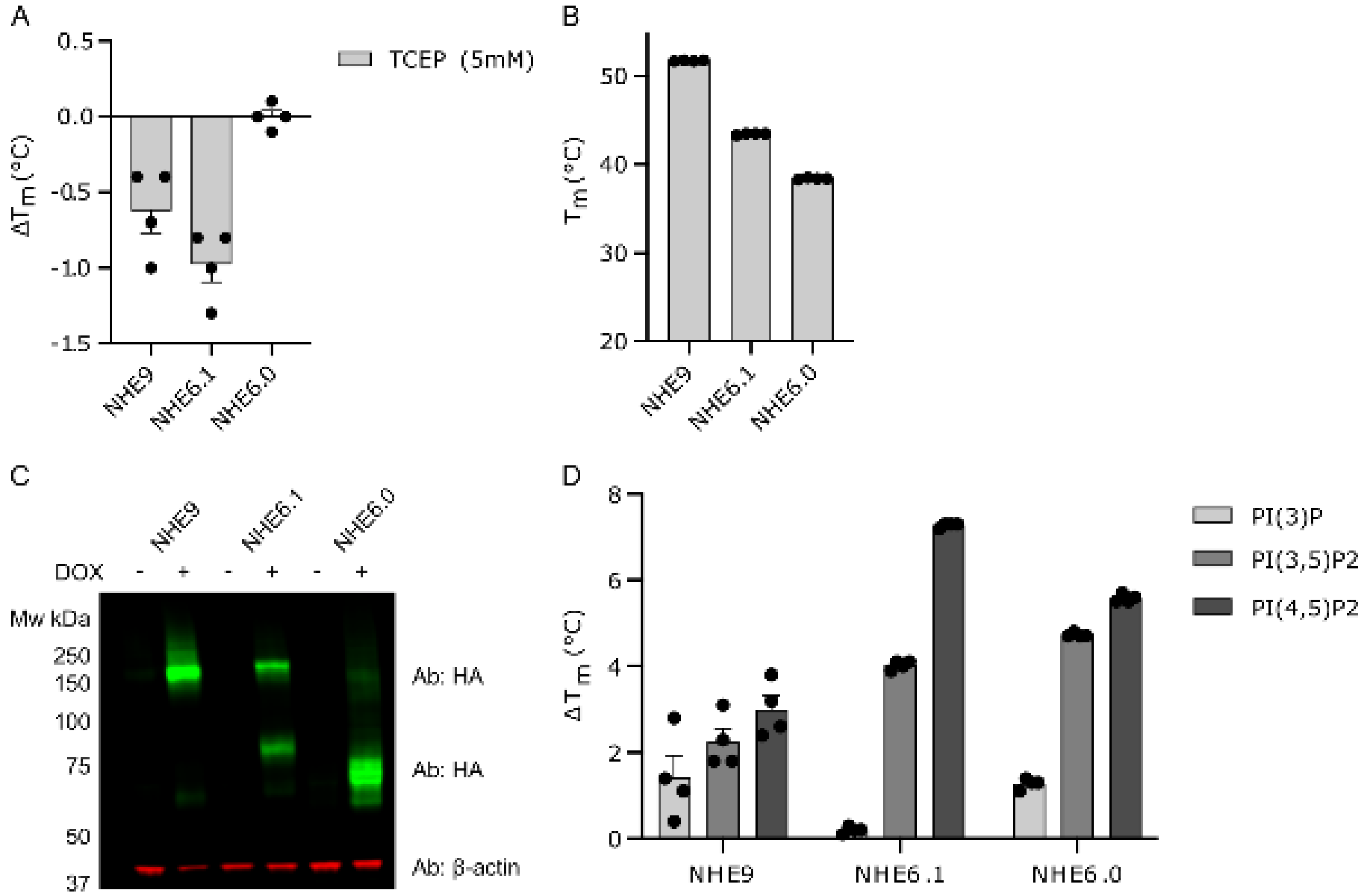

7. *Supplementary Fig. 10 – there is a typo in the last sentence NH6.1 – should be NHE6.1*

We thank the reviewer for spotting this and have corrected the text.

8. *Is anything known under which conditions either of the isoforms NHE6.0 vs. NHE6.1 is expressed? These data can be included in the discussion to enhance the manuscript's physiological relevance. Is there any physiological explanation why NHE6.0 is monomeric, and NHE6.1 forms a dimer?*

We thank the reviewer for this excellent question regarding the differential expression of the two isoforms. To our knowledge, the mechanism has not yet been elucidated. We believe both isoforms exist as dimers, consistent with all NHE structures determined to date. However, the reduced dimer stability of NHE6.0 in detergent may indicate activity or regulatory differences between the isoforms. We have revised the discussion to clarify this point:

"Western blot analysis of cell lysates revealed NHE6.1 is partially resistant to SDS, with monomer and dimer species, while NHE6.0 is primarily monomeric indicating it has a less stable dimeric configuration (Supplementary Figure 8C)."

9. *The chapter Structural basis of RACK1 binding NHE6 – the results should be supported by a valid model of direct interaction between obtained structure of NHE6 and RACK1, which of the isoform is on Fig. 3?*

We thank the reviewer for highlighting this. While RACK1 binding broadly to this region has been previously established, there is not enough data to model this interaction with high confidence. Accordingly, we have rephrased the subheading and revised the text to clarify that our data provides insights into the architecture of the cytoplasmic domain and not this interaction:

"**Cytoplasmic domain architecture of NHE6**

While the TMD is conserved among the different mammalian NHE isoforms, the C-terminal domain (CTD) exhibits significant sequence divergence that is thought to enable isoform specific regulation (Supplementary Figure 10). The scaffolding protein RACK1 is known to directly interact with the proximal CTD of organellar NHEs, including NHE6 and NHE9[30]."

Focused experiments using both NHE6 and RACK1 proteins will be needed to define this interaction but are beyond the scope of this study.

10. *Resolved structures show sodium binding to the residue Asp244, even at pH 7.5, where Asp is expected to be protonated. How can this fact influence the stoichiometry of the transport? This should be discussed.*

We thank the reviewer for this comment and would like to refer to the related comment by Reviewer #1. This is a technical limitation for cryo-EM that hopefully future studies will be able to address.

11. *What is known about the binding of inhibitors to the proteins studied? Could the authors support their concluding statement in the last paragraph of Discussion with a model of the interaction of NHE6/NHE9 with an inhibitor?*

We appreciate the reviewer's comment, which is highly relevant to NHE drug discovery. To our knowledge, there are no validated chemical probes for NHE6/NHE9. We decided not to elaborate on this subject as the only available structural data is from the previously published NHE1-cariporide complex, where low sequence identity limits the extent to one can hypothesize about how potential inhibitors might act on NHE6 and NHE9.
